# Nitrophenylpiperazine derivatives as novel tyrosinase inhibitors: design, synthesis, and in silico evaluations

**DOI:** 10.1186/s13065-024-01167-6

**Published:** 2024-04-05

**Authors:** Mehdi Asadi, Fahime Fayazi, Aida Iraji, Reyhaneh Sabourian, Homa Azizian, Mannan Hajimahmoodi, Bagher Larijani, Mohammad Mahdavi, Massoud Amanlou

**Affiliations:** 1 Department of Medicinal Chemistry, School of Pharmacy, Iran University of Medical Sciences, Tehran, Iran; 2 Department of Medicinal Chemistry, Faculty of Pharmacy, Tehran University of Medical Sciences, Tehran, Iran; 3 Stem Cells Technology Research Center, Shiraz University of Medical Sciences, Shiraz, Iran; 4 Central Research Laboratory, Shiraz University of Medical Sciences, Shiraz, Iran; 5 Drug and Food Control Department, Faculty of Pharmacy, Tehran University of Medical Sciences, Tehran, Iran; 6 Endocrinology and Metabolism Research Center, Endocrinology and Metabolism Clinical Sciences Institute, Tehran University of Medical Sciences, Tehran, Islamic Republic of Iran; 7 Experimental Medicine Research Center, Tehran University of Medical Sciences, Tehran, Iran

**Keywords:** Docking studies, Kinetic evaluation, Tyrosinase inhibitors, Nitrophenylpiperazine, Synthesis

## Abstract

A novel series of 4-nitrophenylpiperazine derivatives (**4a**–**m**) was designed and synthesized as potential tyrosinase inhibitors. Comprehensive characterization using ^1^H-NMR, ^13^C-NMR, CNH, and IR techniques was performed for all target compounds. Subsequently, the derivatives were evaluated for their inhibitory activity against tyrosinase. Among them, compound **4l**, featuring an indole moiety at the N−1 position of the piperazine ring, exhibited a significant tyrosinase inhibitory effect with an IC_50_ value of 72.55 μM. Enzyme kinetics analysis revealed that **4l** displayed mixed inhibition of the tyrosinase enzymatic reaction. Molecular docking was carried out in the enzyme’s active site to further investigate the enzyme-inhibitor interactions. Based on the findings, compound **4l** shows promise as a lead structure for the design of potent tyrosinase inhibitors. This study paves the way for the development of more effective tyrosinase inhibitors for potential applications in various fields.

**Supplementary Information:**

The online version contains supplementary material available at 10.1186/s13065-024-01167-6.

## Introduction

Melanin is a critical biopolymer that significantly determines the color of mammalian skin, eyes, and hair. Beyond its pigmentation function, melanin serves as a protective shield, safeguarding skin cells from the harmful effects of UV radiation and eliminating reactive oxygen species, which are damaging byproducts of cellular processes [1]. Melanin is synthesized in the specialized organelles called melanosomes in melanocytes. However, the aberrant production and accumulation of melanin in the face and neck usually cause common pigmentary disorders, such as melasma and post-inflammatory hyperpigmentation, Parkinson’s, Ochronosis, and Alkaptonuria [2, 3].

Tyrosinases (TYRs, EC 1.14.18.1) are widely distributed enzymes found in various organisms, including bacteria, fungi, insects, plants, and mammals. They play a crucial role in regulating the synthetic pathway of melanin formation, a process involved in pigmentation and other essential biological functions. X-ray structures of tyrosinase showed that the enzyme from different origins is a type-3 metalloenzyme with a conserved catalytic domain comprising six histidine residues and two copper ions (CuA and CuB) [4, 5]. Tyrosinases are the key enzyme that catalyzes the conversion of the substrate tyrosine into the intermediate products l-dopa and *o*-quinone (dopaquinone), which is further oxidized into eumelanin and pheomelanin through other interrelated enzymatic and non-enzymatic reactions [6]. As a result, tyrosinase inhibition is a crucial strategy to control melanin synthesis; therefore, tyrosinase inhibitors have gained interest in therapies for skin disorders associated with abnormal pigmentation and dermo-cosmetic treatments [7].

Available tyrosinase inhibitors such as kojic acid, hydroquinone, and arbutin are anti-melanin agents, but they exhibit various adverse side effects, such as contact dermatitis, irritation, leukoderma, and hypochromic [8]. Therefore, it is of great interest for medical and cosmetic applications to synthesize novel inhibitors. Over the past few years, different tyrosinase inhibitors have been developed, including azole and thiazolidine, thiosemicarbazones, quinone, xanthate, and carboxylic acids [6].

**Fig. 1:**
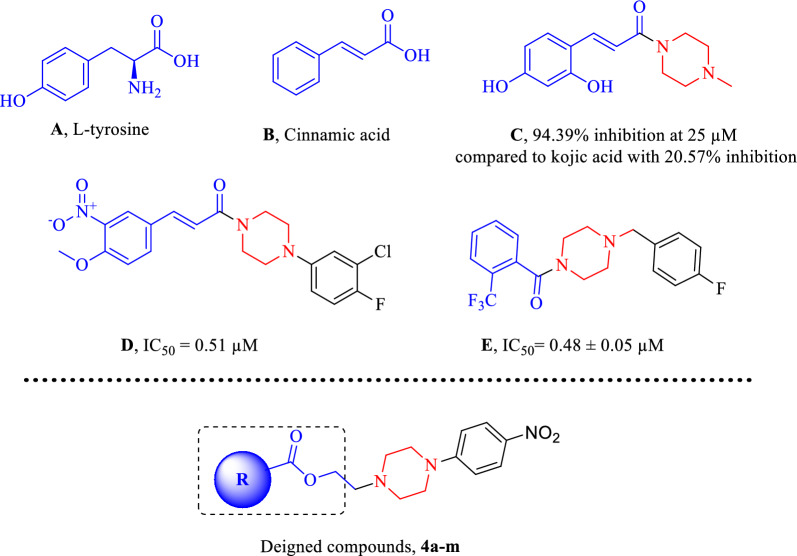
Potent inhibitors of mushroom tyrosinase from previous studies and newly designed compounds (**4a**–**m)**

Tyrosine (compound **A**, Fig. 1) is the natural substrate of tyrosinase enzyme, and a variety of inhibitors mimic the chemical structure of tyrosinase’s natural substrate to hinder the oxidation process of tyrosinase [9]. Cinnamic acid, as an organic acid with low toxicity (compound **B**), presents naturally in plants and has been under investigation for a long time due to its therapeutic relevance [10, 11]. This α,β-unsaturated carbonyl structure demonstrated good tyrosinase inhibition. Also, variation of its structural properties exhibited a wide range of tyrosinase inhibitory potentials, from almost inactive, such as caffeic acid, and 3,4-dihydroxycinnamic acid, to highly potent inhibitors [12]. In the year 2019, cinnamamides, through the conversion of the carboxylic acid functional group into an amido group to increase lipophilicity, were designed. The structure–activity relationship (SAR) showed that *N*-methyl piperazine (compound **C**) had higher activity than morpholine, cyclopentamine, and cyclohexylamine derivatives. Analysis of tyrosinase activity and melanin content in B16F10 cells showed that compound **C** dose-dependently inhibited both cellular tyrosinase activity and melanin content [13]. Also, it was demonstrated that derivatives with heteroatom capable of H-bound interaction group on the aromatic ring of the scaffold generally showed high tyrosinase inhibitory activities [14]. Romagnoli et al. also developed cinnamic acid derivatives linked to aryl piperazines (compound **D**) in 2022. It was shown that the piperazine ring could provide the proper balance between flexibility and rigidity to correctly orientate the substituted moiety into the active site of tyrosinase. As reported by the previous study, the presence of electron-withdrawing substituent (–NO_2_) and electron-releasing substituent (–OCH_3_) capable of H-bound interaction with enzyme improves the inhibition [15]. In another study, a series of 1-(4-fluorobenzyl)piperazine (compound **E**) fragments were introduced as potent tyrosinase inhibitors with IC_50_ values in the range of 0.48–14.66 µM [16].

In this study, we utilized the structural similarity between l-tyrosine and the previously reported potent derivatives by incorporating aryl substitutions linked to nitrophenyl piperazine. This approach was explored as a potential strategy for designing new tyrosinase inhibitors. After synthesizing all the compounds, their tyrosinase inhibition was evaluated using tyrosinase. The most potent derivative from this group was then selected for further investigation through kinetic and molecular docking studies to better understand its inhibitory properties and binding interactions with the enzyme.

## Results and discussion

### Chemistry

**Scheme 1:**
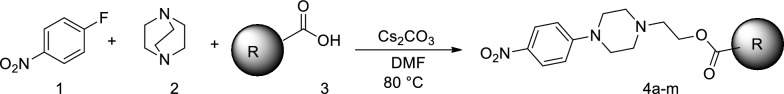
Synthesis of 4-nitrophenyl piperazine derivatives **4a**–**m**

**Scheme 2:**
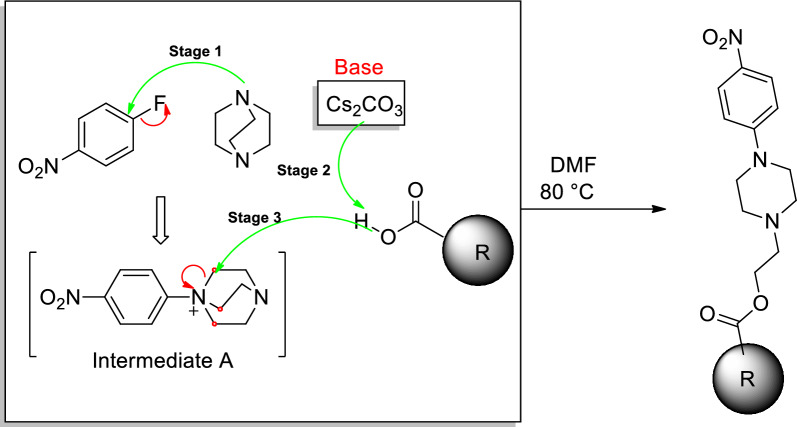
Mechanism of 4-nitrophenyl piperazine derivatives **4a**–**m**

The synthesis and mechanism of the compounds 4a–m were carried out during a **one**-**pot** stepwise synthesis (OPSS) that, were schematically described in Schemes 1 and 2 respectively. As shown in the mechanism of this reaction, in the first stage, DABCO attacked 4-fluoronitrobenzene as a nucleophile, which caused the formation of intermediate A. This intermediate has a quaternary amine in its structure and is under pressure due to its bicyclic structure. In the second stage, the cesium carbonate (strong mineral base) absorbs hydrogen of acid derivatives and the resulting carboxylates, as a nucleophile, attack the carbons adjacent to the ammonium group which are in the same position, causing the opening of the bicyclic ring and the release of nitrogen from the positive charge pressure, creating the final products **4a**–**m**. The yield of the final products as mentioned in the spectral information section was obtained in the range of 75–88%, which indicates the cheap and fast synthesis chosen for this reaction. Finally, the structure of final products 4a–m was confirmed using NMR, IR spectroscopy, and elemental analysis.

### Tyrosinase inhibitory activity assay

Tyrosinase inhibitory assay of all synthesized nitrophenyl piperazine derivatives **4a**–**m** was performed using l-dopa as the substrate, and the results are summarized in Table 1.

**Table 1:** Tyrosinase inhibitory activity of nitrophenylpiperazine


Entry	R	% Inhibition at 100 μM^a^	IC_50_ ± SD^a^ (μM)
**4a**	Phenyl	35.8 ± 3.24	174.71 ± 0.68
**4b**	2-Bromophenyl	20.6 ± 2.58	> 200
**4c**	2,4-Dichlorophenyl	27.9 ± 3.09	> 200
**4d**	4-Nitrophenyl	28.3 ± 2.89	203.23 ± 1.16
4e	3-Nitrophenyl	24.9 ± 2.81	> 200
**4f**	2-Chloro-4-nitrophenyl	35.1 ± 2.25	> 200
**4g**	4-Methoxyphenyl	30.0 ± 4.04	> 200
**4h**	2,3-Dimethoxybenzoate	26.9 ± 3.55	200.88 ± 1.32
**4i**	Benzyl	39.4 ± 4.18	184.24 ± 0.88
**4j**	Vinylbenzene	23.1 ± 3.60	> 200
**4k**	3-Pyridine	54.3 ± 4.58	82.68 ± 1.16
**4l**	2-Indole	66.6 ± 4.12	72.55 ± 0.49
**4m**	5-Nitrofuran	43.7 ± 3.18	175.28 ± 0.24
**Kojic acid**^b^	–	–	27.56 ± 1.27

^a^SD (standard deviation)

^b^Positive control

Compound 4a with no substitute group was selected as the template compound exhibited IC_50_ = 174.71 μM. The presence of 2-Br (**4b**, IC_50_ > 200 μM) and 2,4-dichloro (**4c**, IC_50_ > 200 μM) as halogen substitution on the phenyl ring did not improve the potencies compared with **4a**. Also, nitro substituent as a strong electron-withdrawing group at the *para* (**4d**) or *meta* (**4e**) position of the phenyl ring did not empower the potency vs **4a**. Also, 2-chloro-4-nitrophenyl substitution (**4f**) appeared to deteriorate the inhibitory activities (IC_50_ > 200 µM). Incorporating 2,3-dimethoxy benzoate in compound **4h** results in a slight increase in the activity compared to the rest of the halogen-substituted groups.

Next, the elongation of the linker was also evaluated, and it was revealed that compound **4i** bearing benzyl moiety had less potency than the phenyl counterpart, **4a**, with an IC_50_ value of 184.24 µM. Also, the effect of bound unsaturation with the same structure as cinnamic acid was examined. Such substitution in **4j** did not alleviate the activity (IC_50_ > 200 µM).

To better extract the SARs, ring replacement was performed; noteworthy, 3-pyridine substitution (**4k**) significantly improved the potency compared with phenyl and benzyl counterparts with IC_50_ value of 82.68 µM. The best results in this set of compounds came back to **4l** bearing indole moiety at R position with IC_50_ = 72.55 µM. In contrast, **4m** bearing 5-nitrofuran reduced the activity compared to **4k** and **4l**; it appeared to have lower inhibitory activities than most compounds.

In summary, the presence of phenyl or benzyl at the R position did not show significant inhibition, and any substitutions on this ring were also unfavorable for tyrosinase inhibition. However, replacing benzyl with 3-pyridine or 2-indole led to a notable improvement in potency compared to the rest of the derivatives. These findings highlight the importance of specific substitutions at the R position in enhancing the inhibitory activity of the compounds against tyrosinase.

### Inhibition mechanism

**Fig. 2:**
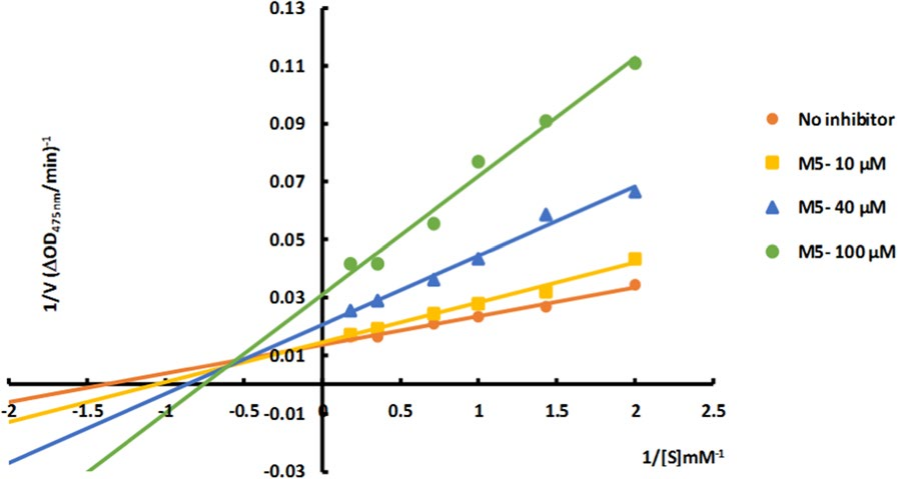
Lineweaver–Burk plot for the inhibition of mushroom tyrosinase catalyzed l-dopa oxidation by **4l**

The most potent derivative, compound **4l**, was studied for its enzyme inhibition mode using Lineweaver–Burk plot analysis. The results, depicted in Fig. 2 and summarized in Table 2, showed that the *K*_*m*_ and *V*_*max*_ values increased as the inhibitor concentration was raised. The Lineweaver–Burk plots for tyrosinase inhibition with various concentrations of **4l** and l-dopa displayed straight lines intersecting the x-axis at similar points. These findings indicate that compound **4l** acts as a mixed inhibitor against mushroom tyrosinase, affecting substrate binding and enzyme catalysis.

**Table 2:** Kinetic parameters for the compounds **4l** against mushroom tyrosinase inhibition assay

Concentration (µM)	V_max_ (mM/Min)	*K*_*m*_ (mM)
No inhibitor	66.94	0.38
10	65.79	0.48
40	64.10	0.66
100	62.89	0.76

### Molecular docking

**Fig. 3:**
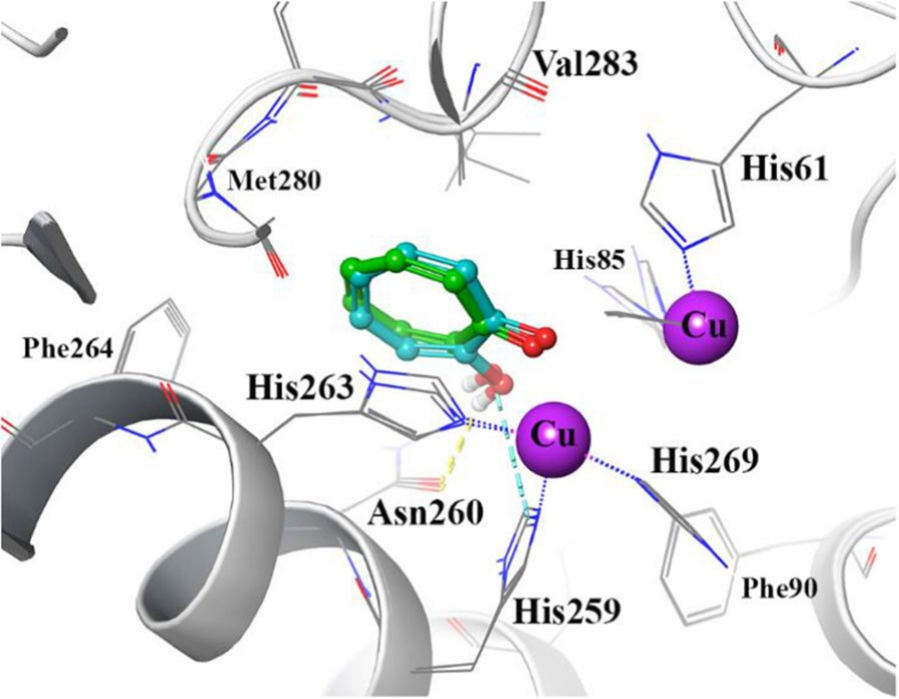
Representation of the tyrosinase active site, the tropolone co-crystallized and the corresponding re-docked form are represented in green and cyan color, respectively

The reliability of the applied docking protocol was assessed by re-docking of tropolone into the active site of the tyrosinase enzyme. The key characteristic of a good docking program is its ability to reproduce the experimental or crystallographic binding modes of ligands. To test this, a ligand is taken out of the X-ray structure of its protein–ligand complex and re-docked into its binding site. The docked binding mode is then compared with the experimental binding mode, and the RMSD is calculated; a prediction of a binding mode is considered successful if the RMSD is below a certain value (usually < 2.0 Å). Figure 3 shows the superimposed structures between the docked and the crystallographic tropolone over tyrosinase active site which its RMSD is in acceptable value within the cutoff limit (1.02 Å). This protocol was then similarly applied to all synthesized compounds (**4a**–**4m**).

To gain insight into the inhibitory activity of compound**s**, molecular docking was performed to investigate its interaction pattern with the active site of tyrosinase. Analysis of the docked ligand poses showed that His61, His85, His259, His263 were the top residues producing the greatest number of interactions at the tyrosinase bi nuclear active site pocket. Table 3 shows docking binding score and the free binding energy calculation based on MM-GBSA calculation of the synthesized compounds (**4a**–**4m**). The obtained MM-GBSA energy are close to and correlated with the mentioned experimental results. Compounds Kojic acid and **4l** with the highest inhibitory activity represent the MM-GBSA energy of − 80.6 and − 76.3 kcal mol^−1^, respectively, while compounds with lowest IC_50_ represent lower MM-GBSA energy (Table 3).

**Table 3:** Docking binding score and the free binding energy calculation based on MM-GBSA calculation of the synthesized compounds (**4a**–**4m**)

Entry	Docking binding score	MM-GBSA energy (Kcal/mol)
**4l**	− 5.43	− 76.3
**4e**	− 5.20	− 63.2
**4f**	− 4.36	− 43.4
**4b**	− 5.12	− 60.12
**4k**	− 5.31	− 75.07
**4d**	− 4.99	− 61.3
**4m**	− 5.01	− 60.24
**4j**	− 4.81	− 47.9
**4c**	− 5.67	− 59.46
**4g**	− 4.83	− 61.8
**4i**	− 5.16	− 65.41
**4a**	− 5.25	− 62.95
**4h**	− 4.36	− 54.7
**Kojic acid**	− 6.74	− 80.6

**Fig. 4:**
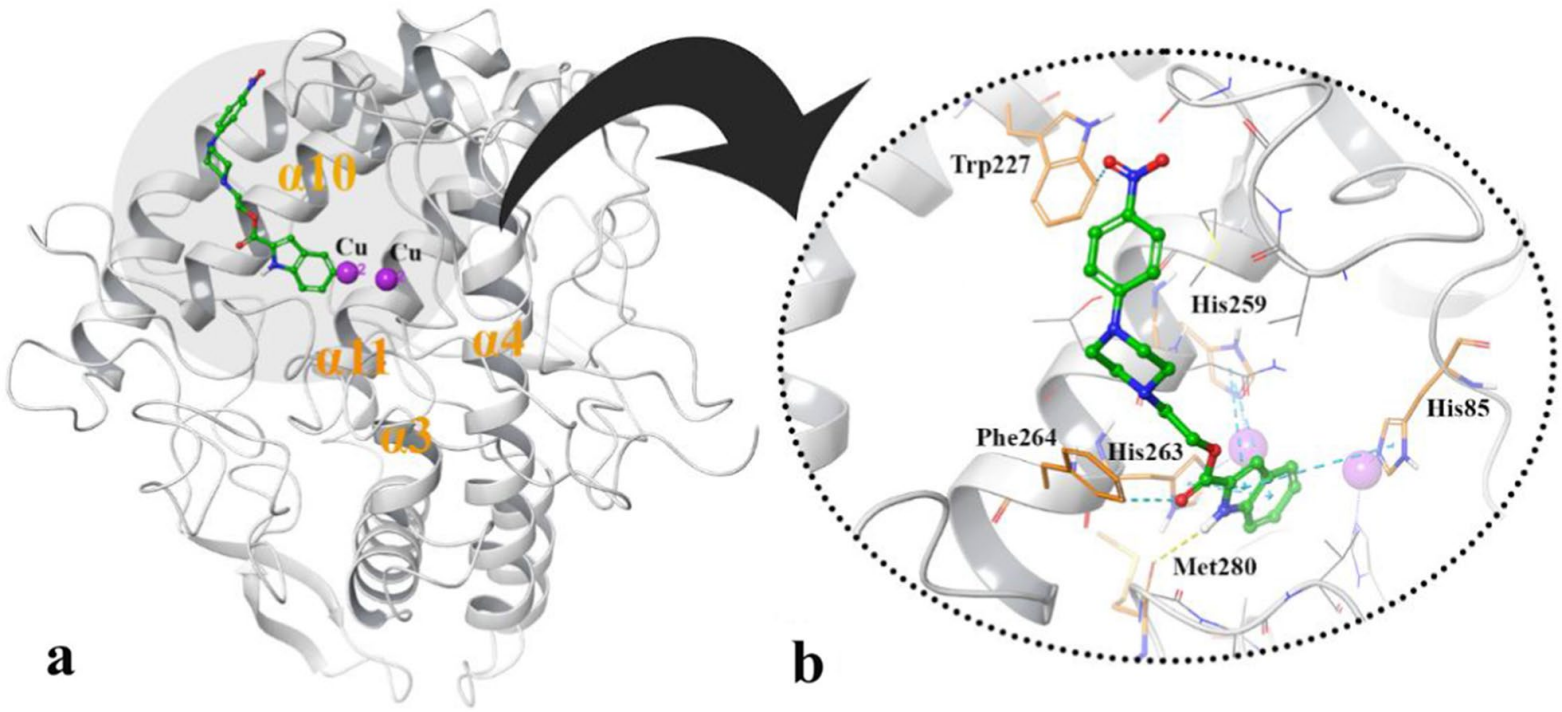
Representation of the compounds docking poses over the tyrosinase enzyme (**a**) close-up illustration and the non-boding interaction pattern of compound **4l** over the tyrosinase active site (**b**)

Docking study of compound **4l** showed that it formed deep interactions with the active site residues and di-copper ions which are surrounded by α3/α4 and α10/α11 helices over the active pocket of tyrosinase enzyme (Fig. 4a). Also, Fig. 4b shows that the **4l** indole group orients toward the two-Copper ions through several hydrophobic pi-pi interactions with the imidazolidine rings of His85, His259 and His263, which coordinate to the di-copper core of the active site. Additionally, the indolyl NH group provides H-bond interaction with the backbone carbonyl group of Met280. Additionally, Trp227 and Phe264 are the remaining residues for stabilizing compound **4l** over the active site pocket through hydrophobic interaction with the ester linker and the nitro group of the mentioned compound.

### Molecular dynamic (MD) simulation

In order to study the stability of the complex, the best induced fit docked pose of compound 4l was implemented as starting points for 150 ns MD simulation in order to predict the motion and the dynamic behavior of complexed systems at an atomistic level [17].

Root mean square deviation (RMSD) values are indicative of the conformational stability and perturbations of system. When RMSD values no longer follow a specific trend but fluctuate around a certain point it can be argued that the complex has reached equilibrium [18].

**Fig. 5:**
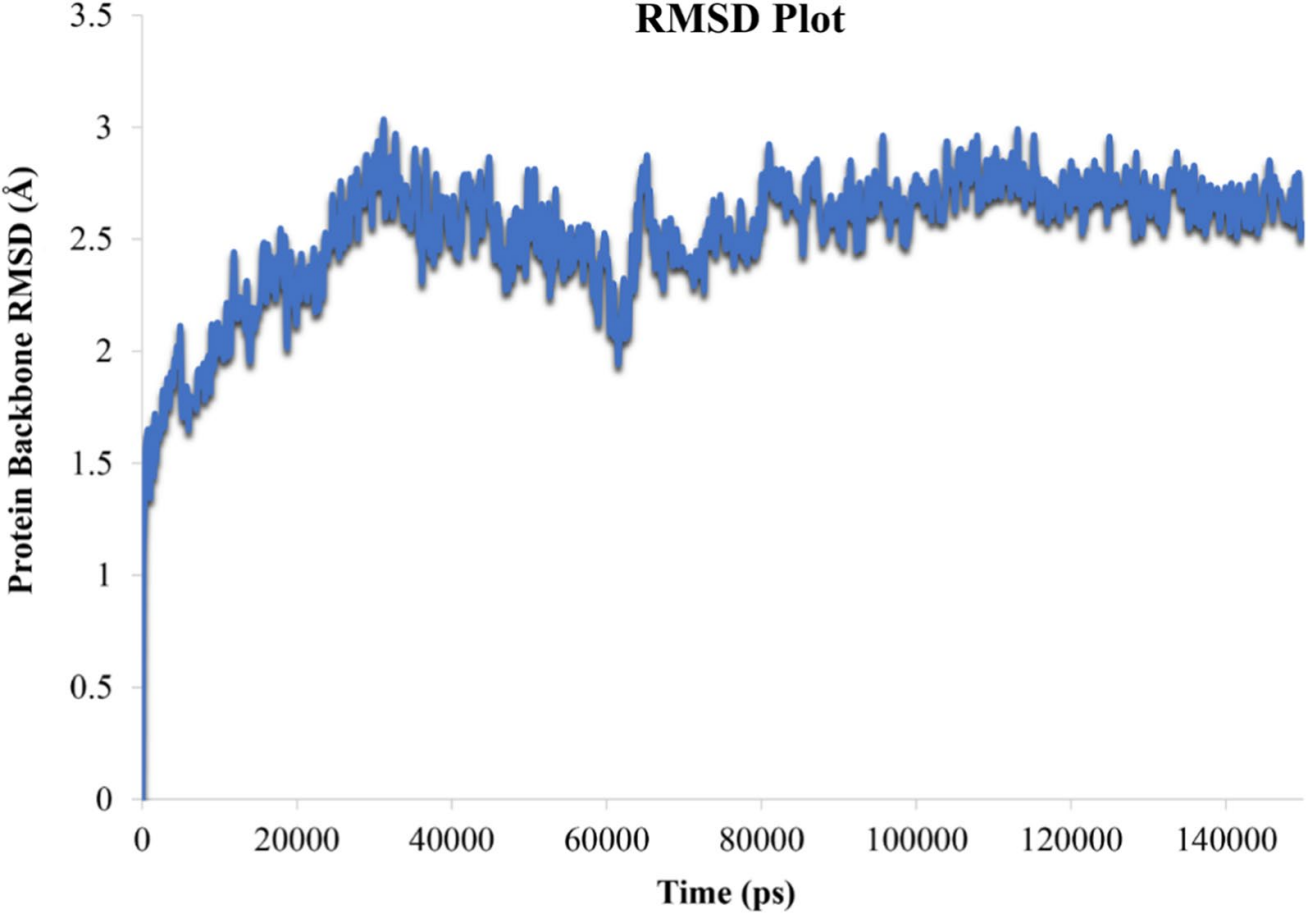
RMSD plot of tyrosinase backbone in complex with compound **4l**, throughout the 150 ns of the simulation time

Figure 5 depicts the protein backbone RMSD values for the tyrosinase-4l complex over about 150 ns MD simulation time. It is obvious that the RMSD value of the tyrosinase complexed with compound 4l fluctuate through the first 80 ns and reaches to an equilibration for the rest of simulation time. Such observation indicated that the employed simulation time was enough to obtain an equilibrium structure over the simulation time.

**Fig. 6:**
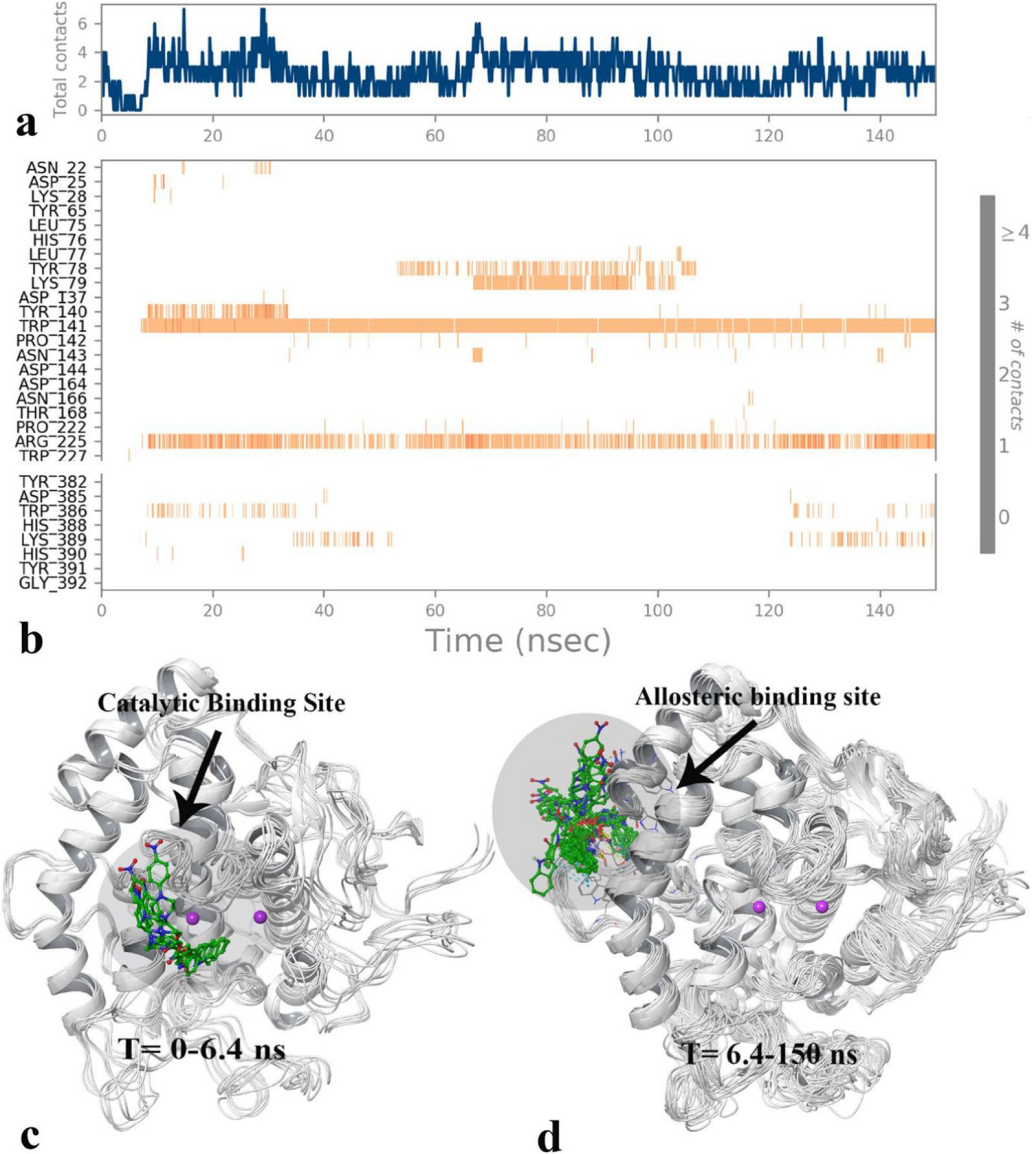
The total tyrosinase residues contact (**a**) and the timeline representation of the interactions of compound **4l** in each trajectory frame during the total MD simulation time (the more contact of ligand with residues, the darker shade of orange color) (**b**). The 3D representation of tyrosinase with compound **4l** in two different binding pockets related to 0–6.4 ns (**c**) and 6.4 to the rest of simulation time (**d**) (Desmond v5.3)

The total contact diagram of MD procedure revealed that, the quantity of ligand-tyrosinase interaction decreased from 4 contacts at the beginning of the MD simulation to zero during the short period time and after that it raised and fluctuated between 2 and 6 contacts for the majority of the MD simulation time (Fig. 6a). Also, the mentioned phenomena can be tracked by Fig. 6b in which at a short period time of MD simulation time there are few ligand–residue interaction while after that time it increased (dark orange color) and stabilized for the rest of the MD simulation time.

Additionally, the visual inspection of the MD trajectories revealed that the MD simulation time divided in two sections; the first part with short duration was from 0 to 6.4 in which compound **4l** located at the heart of the active site and oriented towards the di-copper catalytic active site through its indoline moiety (Fig. 6c). Otherwise the other one belongs to the significant amount of time last from 6.4 to 150 ns in which the ligand resided to the secondary binding pocket which was far from the bi-metal active site and which faced toward the final C-terminal helix of the tyrosinase enzyme (Fig. 5d). The new pose of compound **4l** increased an average number of ligand-enzyme contacts from 0 to about 4–6 interactions (Fig. 6a).

**Fig. 7:**
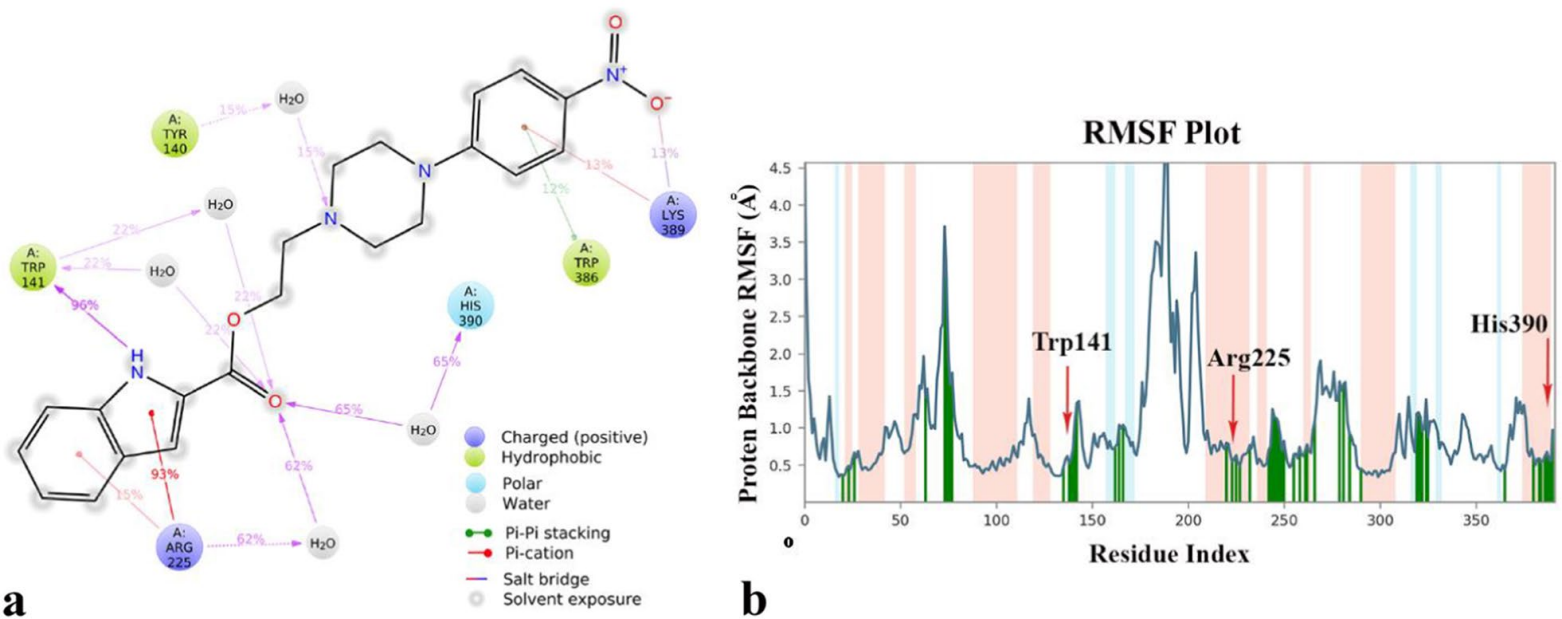
2D interaction diagram of compound **4l-**tyrosinase complex which is responsible for through the whole MD simulation time (**a**), RMSF plot of the tyrosinase residue in complexed with compound **4l** over 150 ns MD simulation time (**b**) α-helical and ß-strand regions are highlighted in light pink and blue backgrounds, respectively

Furthermore, the 2D interactions diagram of compound **4l**-tyrosinase complex in with the enzyme are depicted in Fig. 7a shows that the aromatic property of the indoline ring and corresponding –NH group stabilized with Arg225 and Trp141 through the direct hydrogen bond and pi-cation interaction for about the majority of the MD simulation time (93% and 95% of the simulation time, respectively). Also, the carbonyl part of the ester group interacted through water mediated hydrogen bond by His390 for about 35% of MD simulation time. Also, the nitrogen atom belongs to the piperazine ring provided indirect H-bond interaction with Tyr140 for about 15% and finally the *p*-nitro phenyl group provided the occasionally salt-bridge, Pi-cation and pi–pi stacking interaction with Trp386 and Lys389 at the C-terminal domain of the enzyme. Previously, Tyr140 and the correspond environment introduced as allosteric binding site of tyrosinase enzyme [19]. Based on the RMSD plot it can be revealed that the mentioned ligand interactions caused decreasing the corresponding residue fluctuation at different domain of the enzyme (Fig. 7b).

In summary, through MD simulation investigation, it is revealed that the resulted IFD posed of compound **4l** is slightly stay at the bi-metal active site and stable at the allosteric secondary binding pocket which is far from the active site and is responsible for many important interactions. Based on enzyme kinetic studies, compound **4l** exerted a mixed-type inhibition, binding ability of **4l** with both the catalytic site and the allosteric sites confirmed its mixed-type inhibition of tyrosinase.

### In silico prediction of pharmacokinetic properties

In order to predict the oral and gastrointestinal absorption of compound **4l** and **4k** as higher biological activated compound in this series and kojic acid as standard inhibitor of tyrosinase, the predicted aqueous solubility (Log *S*), the predicted apparent Caco-2 cell permeability as a model for the gut-blood barrier (non-active transport) (LogCaco-2) and the predicted % human intestinal absorption (%HIA) calculated with the aid of pkCSM web server (http://biosig.unimelb.edu.au/pkcsm).

According to Table 4, the predicted solubility of all compounds are in favorable value. In this all compound exhibited high HIA in which their value is higher than 30%.

**Table 4:** Predicted physico-chemical and absorption activity of compound 4k, 4l and kojic acid

Compound	Mw	LogP	Log S^a^	Log Caco-2^b^	%HIA^c^
**4k**	356	1.96	− 3.394	0.97	94.3
**4l**	394.16	3.0	− 3.823	0.259	90.671
**Kojic acid**	142.1	− 0.162	− 1.69	0.64	88

^a^Predicted aqueous solubility in mol L^−1^ (− 6.5 to 0.5) (QPlogS > − 5.7)

^b^Predicted Caco-2 cell permeability of a given compound is given as the log Papp in 10^−6^ cm/s (high Caco-2 permeability has LogCaco-2 > 0.9)

^c^Percent of human intestinal absorption, (< 30% is poor and > 30% is high)

## Conclusion

In conclusion, this study designed, synthesized, and screened nitrophenyl piperazine derivatives with aryl substitutions against tyrosinase. Among them, compound **4l** containing an indole derivative exhibited the highest potency with an IC_50_ value of 72.55 µM. The SAR analysis revealed that replacing the benzyl or phenyl ring with indole or pyridine moiety significantly improved the potencies of the compounds. Kinetic studies of the most potent derivative indicated mixed inhibition against tyrosinase. Molecular docking studies provided insights into the key interactions, including hydrogen bonding, π–π interactions, and salt bridge interactions, between tyrosinase and compound **4l**. These findings suggest that nitrophenylpiperazine derivatives hold promise as potential anti-tyrosinase agents for applications in medicine, agriculture, and the food industry.

## Materials and methods

### Synthesis

#### General synthesis procedure of 4-nitrophenyl piperazine derivatives 4a–m

Synthesis of the compounds **4a**–**m**. There is a conventional reaction between DABCO and 4-fluoronitrobenzene led to the formation of A mixture of 4-fluoronitrobenzene (compound **1**, 1 mmol), DABCO (compound **2**, 1.2 mmol), benzoic acid derivatives (compound **3**, 1.2 mmol), and Cs_2_CO_3_ (1.3 mmol) in DMF (5 ml) was stirred at 80 °C for 4 h. A stopwatch monitored the time of the reaction. The progress of the reaction was monitored by thin-layer chromatography. On completion of the reaction, the reaction mixture was drowned in water. The precipitated product was filtered and dried.

#### Spectral information

##### 2-(4-(4-Nitrophenyl)piperazin-1-yl)ethyl benzoate (4a)

White solid; isolated yield: 75%, M.p: 176–178 °C; IR (ν, cm^−1^): 1729, 1552, 1450, 1348. ^1^H NMR (500 MHz, DMSO-*d*_*6*_) δ 8.00 (dd, *J* = 32.8, 8.6 Hz, 4H), 7.66 (t, *J* = 7.5 Hz, 1H), 7.53 (t, *J* = 7.7 Hz, 2H), 7.01 (d, *J* = 8.9 Hz, 2H), 4.43 (t, *J* = 5.7 Hz, 2H), 3.44 (t, *J* = 5.0 Hz, 4H), 2.77 (t, *J* = 5.7 Hz, 2H), 2.62 (t, *J* = 5.0 Hz, 4H). ^13^C NMR (125 MHz, DMSO-*d*_*6*_) δ 166.13, 155.17, 137.31, 133.78, 130.23, 129.60, 129.56, 129.23, 126.13, 113.05, 112.99, 62.74, 56.34, 52.83, 46.82. *Anal*. Calcd. for C_19_H_21_N_3_O_4_ (355.39): C, 64.21; H, 5.96; N, 11.82. Found: C, 64.11; H, 5.84; N, 11.65 (Additional file 1).

##### 2-(4-(4-Nitrophenyl)piperazin-1-yl)ethyl 2-bromobenzoate (4b)

White solid; isolated yield: 83%, M.p: 185–187 °C; IR (ν, cm^−1^): 1726, 1549, 1354, 1293, 656. ^1^H NMR (500 MHz, DMSO-*d*_*6*_) δ 8.03 (d, *J* = 8.9 Hz, 2H), 7.75 (t, *J* = 7.6 Hz, 2H), 7.49 (p, *J* = 7.6 Hz, 2H), 7.00 (d, *J* = 9.0 Hz, 2H), 4.43 (t, *J* = 5.7 Hz, 2H), 3.43 (t, *J* = 5.0 Hz, 4H), 2.74 (t, *J* = 5.7 Hz, 2H), 2.60 (t, *J* = 5.0 Hz, 4H). ^13^C NMR (125 MHz, DMSO-*d*_*6*_) δ 166.15, 155.15, 137.31, 134.32, 133.49, 133.07, 131.23, 128.30, 126.12, 120.54, 113.04, 62.98, 56.20, 52.75, 46.82. *Anal*. Calcd. for C_19_H_20_BrN_3_O_4_ (434.28): C, 52.55; H, 4.64; N, 9.68. Found: C, 52.37; H, 4.85; N, 9.53.

##### 2-(4-(4-Nitrophenyl)piperazin-1-yl)ethyl 2,4-dichlorobenzoate (4c)

White solid; isolated yield: 87%, M.p: 196–198 °C; IR (ν, cm^−1^): 1741, 1549, 1428, 1347, 1185, 773. ^1^H NMR (400 MHz, DMSO-*d*_6_) δ 8.07–8.00 (m, 2H), 7.84 (d, *J* = 8.4 Hz, 1H), 7.78 (d, *J* = 2.0 Hz, 1H), 7.57 (dd, *J* = 8.4, 2.1 Hz, 1H), 7.06–6.97 (m, 2H), 4.44 (t, *J* = 5.6 Hz, 2H), 3.43 (t, *J* = 5.1 Hz, 4H), 2.74 (t, *J* = 5.6 Hz, 2H), 2.60 (t, *J* = 5.1 Hz, 4H). ^13^C NMR (100 MHz, DMSO-*d*_6_) δ 164.64, 155.16, 137.62, 137.28, 133.61, 132.93, 130.84, 129.42, 128.18, 126.15, 113.05, 63.10, 56.15, 52.72, 46.79. *Anal*. Calcd. for C_19_H_19_Cl_2_N_3_O_4_: C, 53.79; H, 4.51; N, 9.90. Found: C, 53.61; H, 4.72; N, 9.80.

##### 2-(4-(4-Nitrophenyl)piperazin-1-yl)ethyl 4-nitrobenzoate (4d)

Light brown solid; isolated yield: 87%, M.p: 172–174 °C; IR (ν, cm^−1^): 1736, 1556, 1343, 1210. ^1^H NMR (400 MHz, DMSO-*d*_6_) δ 8.41–8.29 (m, 2H), 8.25–8.13 (m, 2H), 8.12–7.98 (m, 2H), 7.01 (d, *J* = 9.4 Hz, 2H), 4.49 (t, *J* = 5.6 Hz, 2H), 3.44 (t, *J* = 5.1 Hz, 4H), 2.79 (t, *J* = 5.6 Hz, 2H), 2.63 (t, *J* = 5.1 Hz, 4H). ^13^C NMR (100 MHz, DMSO-*d*_6_) 164.72, 155.14, 150.67, 137.27, 135.61, 131.09, 126.15, 124.38, 113.03, 63.41, 56.19, 52.77, 46.78. *Anal*. Calcd for C_19_H_20_N_4_O_6_: C, 57.00; H, 5.04; N, 13.99. Found: C, 56.81; H, 5.19; N, 13.83.

##### 2-(4-(4-Nitrophenyl)piperazin-1-yl)ethyl 3-nitrobenzoate (4e)

Light brown solid; isolated yield: 84%, M.p: 181–183 °C; IR (ν, cm^−1^): 1731, 1558, 1348, 1224. ^1^H NMR (400 MHz, DMSO-*d*_6_) δ 8.62 (t, *J* = 2.0 Hz, 1H), 8.50 (ddd, *J* = 8.3, 2.4, 1.2 Hz, 1H), 8.37 (dt, *J* = 7.8, 1.3 Hz, 1H), 8.11–7.95 (m, 2H), 7.85 (t, *J* = 8.0 Hz, 1H), 7.10–6.90 (m, 2H), 4.50 (t, *J* = 5.7 Hz, 2H), 3.44 (t, *J* = 5.1 Hz, 4H), 2.80 (t, *J* = 5.7 Hz, 2H), 2.63 (t, *J* = 5.1 Hz, 4H). ^13^C NMR (100 MHz, DMSO-*d*_6_) δ 164.59, 155.51, 150.53, 137.39, 135.27, 131.79, 129.15, 128.35, 126.58, 123.32, 113.35, 63.03, 55.95, 52.61, 46.95. *Anal*. Calcd for C_19_H_20_N_4_O_6_: C, 57.00; H, 5.04; N, 13.99. Found: C, 56.84; H, 5.26; N, 14.14.

##### 2-(4-(4-Nitrophenyl)piperazin-1-yl)ethyl 2-chloro-4-nitrobenzoate (4f)

Light brown solid; isolated yield: 88%, M.p: 203–205 °C; IR (ν, cm^−1^): 1736, 1559, 1358, 1236, 756. ^1^H NMR (500 MHz, DMSO-*d*_*6*_) δ 8.39 (d, *J* = 2.0 Hz, 1H), 8.32–8.26 (m, 1H), 8.07–8.01 (m, 3H), 7.07–6.99 (m, 2H), 4.50 (t, *J* = 5.7 Hz, 2H), 3.44 (t, *J* = 4.9 Hz, 4H), 2.76 (t, *J* = 5.7 Hz, 2H), 2.61 (t, *J* = 4.9 Hz, 4H). ^13^C NMR (125 MHz, DMSO-*d*_*6*_) δ 164.48, 155.18, 149.77, 139.08, 137.32, 136.60, 132.31, 126.14, 125.92, 122.97, 113.08, 63.55, 56.10, 52.69, 46.82. *Anal*. Calcd for C_19_H_19_ClN_4_O_6_ (434.83): C, 52.48; H, 4.40; N, 12.88. Found: C, 52.31; H, 4.57; N, 12.72.

##### 2-(4-(4-Nitrophenyl)piperazin-1-yl)ethyl 2-(4-methoxyphenyl)acetate (4g)

White solid; isolated yield: 77%, M.p: 207–209 °C; IR (ν, cm^−1^): 1734, 1555, 1357, 1286. ^1^H NMR (500 MHz, DMSO-*d*_*6*_) δ 8.05 (d, *J* = 9.4 Hz, 2H), 7.19 (d, *J* = 8.6 Hz, 2H), 7.00 (d, *J* = 9.4 Hz, 2H), 6.87 (d, *J* = 8.6 Hz, 2H), 4.17 (t, *J* = 5.6 Hz, 2H), 3.72 (s, 3H), 3.59 (s, 2H), 3.38 (t, *J* = 5.1 Hz, 4H), 2.58 (t, *J* = 5.7 Hz, 2H), 2.53–2.47 (m, 4H). ^13^C NMR (125 MHz, DMSO-*d*_*6*_) δ 171.77, 158.62, 155.15, 137.31, 130.81, 126.74, 126.14, 114.18, 113.01, 62.09, 56.26, 55.47, 52.75, 46.75, 40.02. *Anal*. Calcd*.* for C_21_H_25_N_3_O_5_ (399.44): C, 63.14; H, 6.31; N, 10.52. Found: C, 63.01; H, 6.20; N, 10.36.

##### 2-(4-(4-Nitrophenyl)piperazin-1-yl)ethyl 6-formyl-2,3-dimethoxybenzoate (4h)

White solid; isolated yield: 78%, M.p: 176–178 °C; IR (ν, cm^−1^): 1723, 1545, 1347, 1258, 1151. ^1^H NMR (500 MHz, DMSO-*d*_*6*_) δ 9.85 (s, 1H), 8.04 (d, *J* = 9.2 Hz, 2H), 7.78 (d, *J* = 8.5 Hz, 1H), 7.36 (d, *J* = 8.5 Hz, 1H), 7.02 (d, *J* = 9.5 Hz, 2H), 4.43 (t, *J* = 5.6 Hz, 2H), 3.96 (s, 3H), 3.77 (s, 3H), 3.43 (t, *J* = 5.0 Hz, 4H), 2.72 (t, *J* = 5.7 Hz, 2H), 2.57 (t, *J* = 5.0 Hz, 4H). ^13^C NMR (125 MHz, DMSO-*d*_*6*_) δ 190.58, 166.17, 157.94, 155.17, 145.88, 137.30, 130.92, 128.70, 126.40, 126.13, 113.90, 113.06, 62.99, 61.76, 56.94, 56.00, 52.69, 46.74.

*Anal*. Calcd. for C_22_H_25_N_3_O_7_ (394.42): C, 59.59; H, 5.68; N, 9.48. Found: C, 59.41; H, 5.89; N, 9.32.

##### 2-(4-(4-Nitrophenyl)piperazin-1-yl)ethyl 2-phenylacetate (4i)

White solid; isolated yield: 84%, M.p: 178–180 °C; IR (ν, cm^−1^): 1736, 1548, 1357, 1199. ^1^H NMR (500 MHz, DMSO-*d*_*6*_) δ 8.06 (d, *J* = 9.4 Hz, 2H), 7.30 (ddd, *J* = 18.2, 12.9, 7.4 Hz, 5H), 7.01 (d, *J* = 9.1 Hz, 2H), 4.19 (t, *J* = 5.7 Hz, 2H), 3.68 (s, 2H), 3.39 (t, *J* = 5.0 Hz, 4H), 2.59 (t, *J* = 5.7 Hz, 2H), 2.51 (d, *J* = 4.8 Hz, 4H). ^13^C NMR (125 MHz, DMSO-*d*_*6*_) δ 171.50, 155.15, 137.31, 134.86, 129.78, 128.78, 127.25, 126.16, 113.05, 62.13, 56.26, 52.75, 46.77, 40.91. *Anal*. Calcd. for C_20_H_23_N_3_O_4_ (369.41): C, 65.03; H, 6.28; N, 11.37. Found: C, 64.88; H, 6.45; N, 11.20.

##### 2-(4-(4-Nitrophenyl)piperazin-1-yl)ethyl cinnamate (4j)

White solid; isolated yield: 77%, M.p: 185–187 °C; IR (ν, cm^−1^): 1720, 1546, 1357, 1288. ^1^H NMR (500 MHz, DMSO-*d*_*6*_) δ 8.04 (d, *J* = 8.7 Hz, 2H), 7.79–7.59 (m, 3H), 7.42 (d, *J* = 5.7 Hz, 3H), 7.01 (d, *J* = 9.0 Hz, 2H), 6.66 (d, *J* = 15.9 Hz, 1H), 4.31 (t, *J* = 5.8 Hz, 2H), 3.44 (t, *J* = 5.0 Hz, 4H), 2.68 (t, *J* = 5.8 Hz, 2H), 2.59 (t, *J* = 4.9 Hz, 4H). ^13^C NMR (125 MHz, DMSO-*d*_*6*_) δ 166.64, 155.17, 145.11, 137.31, 134.43, 130.97, 129.38, 128.86, 126.14, 118.47, 113.05, 61.89, 56.44, 52.85, 46.77. *Anal*. Calcd for C_21_H_23_N_3_O_4_ (381.43): C, 66.13; H, 6.08; N, 11.02. Found: C, 65.98; H, 6.23; N, 11.19.

##### 2-(4-(4-Nitrophenyl)piperazin-1-yl)ethyl nicotinate (4k)

White solid; isolated yield: 77%, M.p: 168–170 °C; IR (ν, cm^−1^): 1723, 1552, 1353. ^1^H NMR (500 MHz, DMSO-*d*_*6*_) δ 9.10 (s, 1H), 8.82 (d, *J* = 4.8 Hz, 1H), 8.29 (d, *J* = 8.0 Hz, 1H), 8.03 (d, *J* = 9.1 Hz, 2H), 7.57 (dd, *J* = 8.0, 4.9 Hz, 1H), 7.01 (d, *J* = 9.0 Hz, 2H), 4.46 (t, *J* = 5.6 Hz, 2H), 3.44 (t, *J* = 4.9 Hz, 4H), 2.78 (t, *J* = 5.6 Hz, 2H), 2.63 (t, *J* = 5.0 Hz, 4H). ^13^C NMR (125 MHz, DMSO-*d*_*6*_) δ 165.13, 155.16, 154.15, 150.45, 137.30, 126.17, 126.13, 124.40, 113.04, 63.07, 56.22, 52.78, 46.82. *Anal*. Calcd for C_18_H_20_N_4_O_4_ (356.38): C, 60.66; H, 5.66; N, 15.72. Found: C, 60.51; H, 5.81; N, 15.58.

##### 2-(4-(4-Nitrophenyl)piperazin-1-yl)ethyl 1*H*-indole-2-carboxylate (4l)

White solid; isolated yield: 81%, M.p: 192–194 °C; IR (ν, cm^−1^): 3360, 1729, 1553, 1350, 1266. ^1^H NMR (500 MHz, DMSO-*d*_*6*_) δ 11.10 (s, 1H), 8.03 (d, *J* = 8.8 Hz, 2H), 7.46 (d, *J* = 7.8 Hz, 1H), 7.37 (d, *J* = 8.1 Hz, 1H), 6.98 (dd, *J* = 14.7, 8.5 Hz, 3H), 6.90 (t, *J* = 7.4 Hz, 1H), 6.53 (s, 1H), 4.45 (t, *J* = 5.7 Hz, 2H), 3.41 (d, *J* = 7.1 Hz, 4H), 2.63 (t, *J* = 5.0 Hz, 2H), 2.53 (t, *J* = 4.9 Hz, 4H). ^13^C NMR (125 MHz, DMSO-*d*_*6*_) δ 164.92, 155.10, 140.48, 137.37, 136.01, 128.50, 126.11, 121.21, 120.86, 118.69, 113.09, 112.34, 101.37, 62.47, 56.44, 52.87, 46.58. *Anal*. Calcd*.* for C_21_H_22_N_4_O_4_ (394.42): C, 63.95; H, 5.62; N, 14.20. Found: C, 63.81; H, 5.77; N, 14.01.

##### 2-(4-(4-Nitrophenyl)piperazin-1-yl)ethyl 5-nitrofuran-2-carboxylate (4m)

Light brown solid; isolated yield: 85%, M.p: 173–175 °C; IR (ν, cm^−1^): 1728, 1560, 1358, 1274. ^1^H NMR (499 MHz, DMSO-*d*_*6*_) δ 8.05 (d, *J* = 9.1 Hz, 2H), 7.79 (d, *J* = 3.9 Hz, 1H), 7.60 (d, *J* = 3.9 Hz, 1H), 7.03 (d, *J* = 8.9 Hz, 2H), 4.49 (t, *J* = 5.6 Hz, 2H), 3.44 (t, *J* = 4.9 Hz, 4H), 2.75 (t, *J* = 5.7 Hz, 2H), 2.62 (t, *J* = 5.0 Hz, 4H). ^13^C NMR (126 MHz, DMSO-*d*_*6*_) δ 157.17, 155.17, 144.51, 137.31, 126.15, 120.47, 113.52, 113.07, 63.49, 56.12, 52.75, 46.78. *Anal*. Calcd for C_17_H_18_N_4_O_7_ (390.35): C, 52.31; H, 4.65; N, 14.35. Found: C, 52.17; H, 4.46; N, 14.19.

### Tyrosinase inhibitory assay

The mushroom tyrosinase activity assays for the synthetic compounds were conducted using a modified procedure from a previous study. In summary, a 96-well microplate was utilized, and 160 μl of phosphate buffer (50 mM, pH 6.8), 10 μl of mushroom tyrosinase (500 U/ml, dissolved in PBS), and 10 μl of the test compound (dissolved in DMSO) were added to each well. Then, 20 μl of l-dopa (7 mM, dissolved in PBS) was added to initiate the enzymatic reaction. The change in absorbance at 475 nm was continuously monitored using a spectrophotometer. DMSO without test compounds was included as a control, while kojic acid was used as the positive control. The IC_50_ value, representing the compound’s inhibitory potency, was determined from dose–response curves of percentage inhibition. All experiments were performed in duplicate, and kojic acid served as the standard control for comparison [20].

### Determination of the inhibition type

The most potent derivative was selected for kinetic analysis, and its inhibitory activity was assessed at inhibitor concentrations of 0, 10, 40, and 100 μM and the substrate (l-dopa) concentrations ranged from 0.5 to 2.8 mM according to the previously reported procedures [21].

### Molecular docking

In order to find out the interaction’s mode of designed molecules over tyrosinase enzyme, Maestro Molecular Modeling platform (version10.5) by Schrödinger, LLC was performed [22]. The X-ray crystal structure of the receptor (PDB ID: 2Y9X) (in complex with tropolone) was obtained from the PDB database [23]. As tyrosinase is reported to have the catalytic active site in H subunit, all the docking studies were performed on H subnit. In addition, prosthetic group and co-factors are not directly involved in tyrosinase inhibition, so they totally removed before docking investigation. Water molecules and co-crystallized ligands were removed from the enzyme’s crystallographic structures. The 2D structures of all synthesized compounds were drawn in Marvin 15.10.12.0 program (http://www.chemaxon.com) [24] and converted into pdb file. The Protein Preparation Wizard [17] and the LigPrep [25] module was used to prepare protein and ligand structure properly. The missing side chains of the proteins were filled using the Prime tool and missing residues were updated.

The accurate side‑chain, backbone conformational changes or both during ligand binding at the active site of tyrosinase enzyme were predicted by IFD method using Glide software (Schrödinger LLC 2018, USA) [26]. The tropolone binding site was used to generate the grid for IFD calculation. The maximum 20 poses with receptor and ligand van der Waals radii of 0.7 and 0.5, respectively considered. Residues within 5 Å of the tropolone at the active site were refined followed by side-chain optimization. Structures whose Prime energy is more than 30 kcal/mol are eliminated based on extra precious Glide docking.

### Molecular dynamic simulation

The X-ray crystallographic structure of tyrosinase (www.rcsb.org) and the structure of the compound with the best tyrosinase inhibition activity used. Molecular simulation was performed using the Desmond v5.3 (Schrödinger 2018‐4 suite) [27].

In order to build the system for MD simulation, the protein–ligand complexe was solvated with SPC explicit water molecules and placed in the center of an orthorhombic box of appropriate size in the periodic boundary condition. Sufficient counter‐ions and a 0.15 M solution of NaCl were also utilized to neutralize the system and to simulate the real cellular ionic concentrations, respectively. The MD protocol involved minimization, pre-production, and finally production MD simulation steps. In the minimization procedure, the entire system was allowed to relax for 2500 steps by the steepest descent approach. Then the temperature of the system was raised from 0 to 300 K with a small force constant on the enzyme in order to restrict any drastic changes. MD simulations were performed via NPT (constant number of atoms, constant pressure i.e., 1.01325 bar and constant temperature i.e. 300 K) ensemble. The Nose–Hoover chain method was used as the default thermostat with 1.0 ps interval and Martyna–Tobias–Klein as the default barostat with 2.0 ps interval by applying isotropic coupling style. Long‐range electrostatic forces were calculated based on particle‐mesh‐based Ewald approach with the he cut‐off radius for columbic forces set to 9.0 Å. Finally, the system subjected to produce MD simulations for 150 ns for each protein–ligand complex. During the simulation every 1000 ps of the actual frame was stored. The dynamic behavior and structural changes of the systems were analyzed by the calculation of the root mean square deviation (RMSD) and RMSF.

### Prime MM-GBSA

The ligand binding energies (ΔG_Bind_) were calculated using molecular mechanics/generalized born surface area (MM-GBSA) modules (Schrödinger LLC 2018) based on the following equation;

$$\Delta {\text{G}}_{{{\text{Bind}}}} = {\text{E}}_{{{\text{Complex}}}} {-}\left[ {{\text{E}}_{{{\text{Receptor}}}} + {\text{E}}_{{{\text{Ligand}}}} } \right],$$

where ΔG_Bind_ is the calculated relative free energy which includes both ligand and receptor strain energy. E_Complex_ is the MM-GBSA energy of the minimized complex, and E_Ligand_ is the MM-GBSA energy of the ligand after removing it from the complex and allowing it to relax. E_Receptor_ is the MM-GBSA energy of relaxed protein after separating it from the ligand.

### Prediction of pharmacokinetic properties

Prediction of the molecular properties of the synthesized compounds were performed using the online servers as pkCSM (http://biosig.unimelb.edu.au/pkcsm/).

### Supplementary Information

**Additional file 1:** The Supplementary Information file contains NMR spectra.

## Data Availability

The datasets used or analyzed during the current study are available from the corresponding authors.
