# Supplementary material for: Nitrophenylpiperazine derivatives as novel tyrosinase inhibitors: design, synthesis, and in silico evaluations
Source: BMC Chem. 2024 Apr 5;18(1):67. doi: 10.1186/s13065-024-01167-6 (PMC10998383; doi:10.1186/s13065-024-01167-6)

**Additional file 1**

**Nitrophenylpiperazine derivatives as novel tyrosinase inhibitors; design, synthesis, and *in silico* evaluations**

**Mehdi Asadi^1^, Fahime Fayazi^2^, Aida Iraji^3,4^, Reyhaneh Sabourian^5^, Homa Azizian^1^, Mannan Hajimahmoodi^5^, Bagher Larijani^6^, Mohammad Mahdavi^6^*, Massoud Amanlou^2,7^***

^1^ Department of Medicinal Chemistry, School of Pharmacy, Iran University of Medical Sciences, Tehran, Iran.

^2^ Department of Medicinal Chemistry, Faculty of Pharmacy, Tehran University of Medical Sciences, Tehran, Iran.

^3^ Stem Cells Technology Research Center, Shiraz University of Medical Sciences, Shiraz, Iran.

^4^ Central Research Laboratory, Shiraz University of Medical Sciences, Shiraz, Iran.

^5^ Drug and Food Control Department, Faculty of Pharmacy, Tehran University of Medical Sciences, Tehran, Iran.

^6^ Endocrinology and Metabolism Research Center, Endocrinology and Metabolism Clinical Sciences Institute, Tehran University of Medical Sciences, Tehran, Islamic Republic of Iran.

^7^ Experimental Medicine Research Center, Tehran University of Medical Sciences, Tehran, Iran.

*Correspondence: Massoud Amanlou, amanlou@tums.ac.ir

**2-(4-(4-nitrophenyl)piperazin-1-yl)ethyl benzoate (4a)**


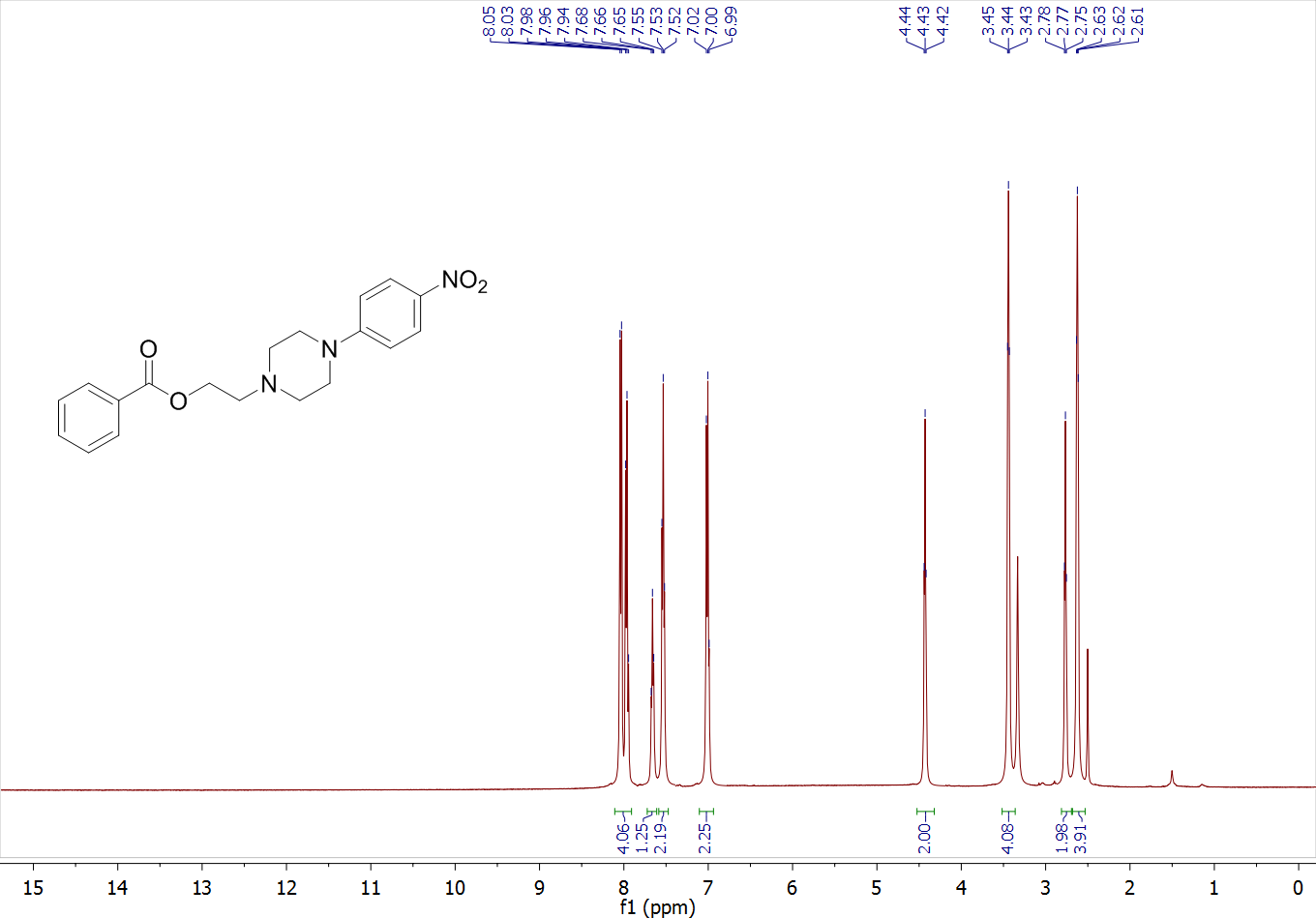


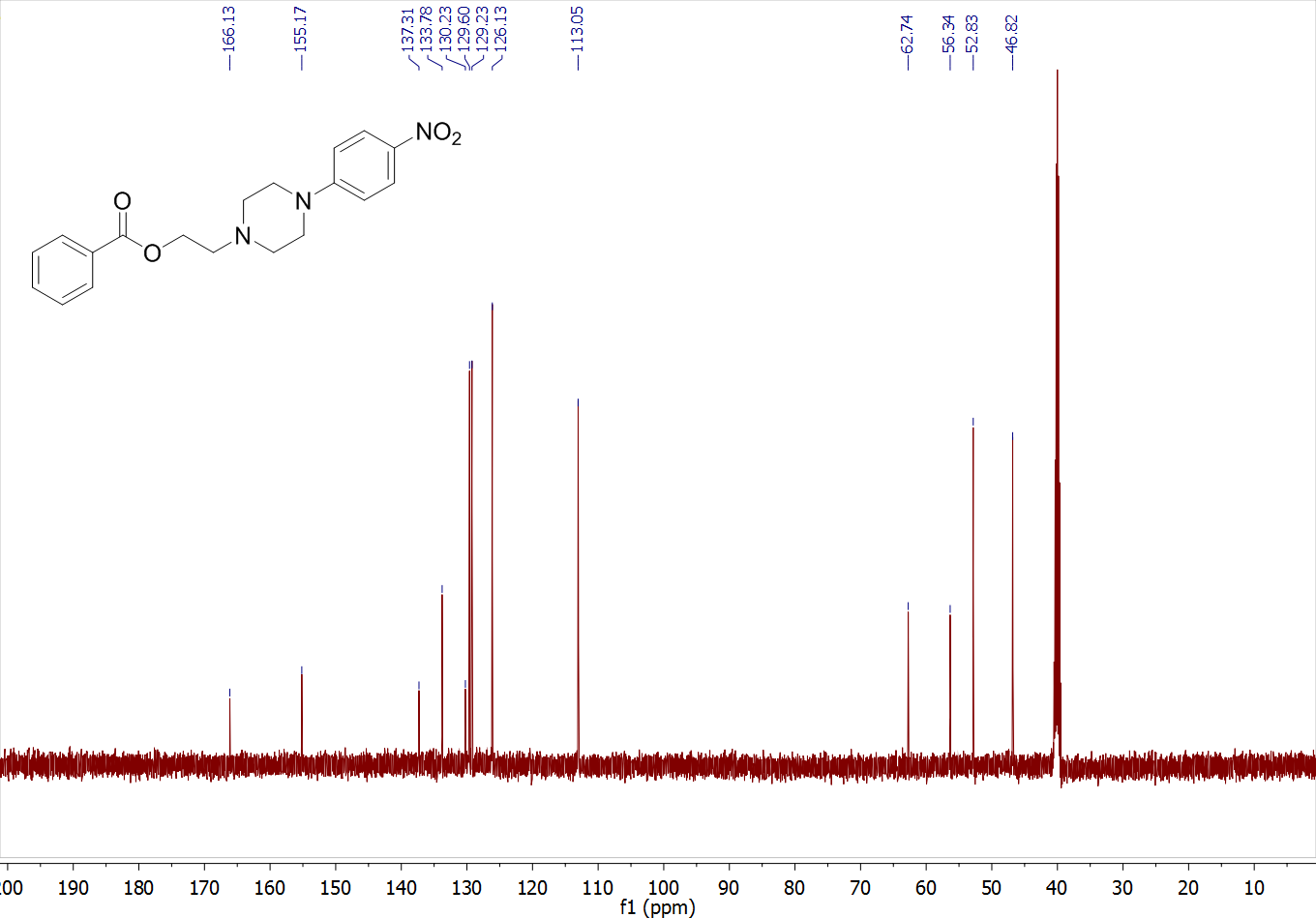


**2-(4-(4-nitrophenyl)piperazin-1-yl)ethyl 2-bromobenzoate (4b)**


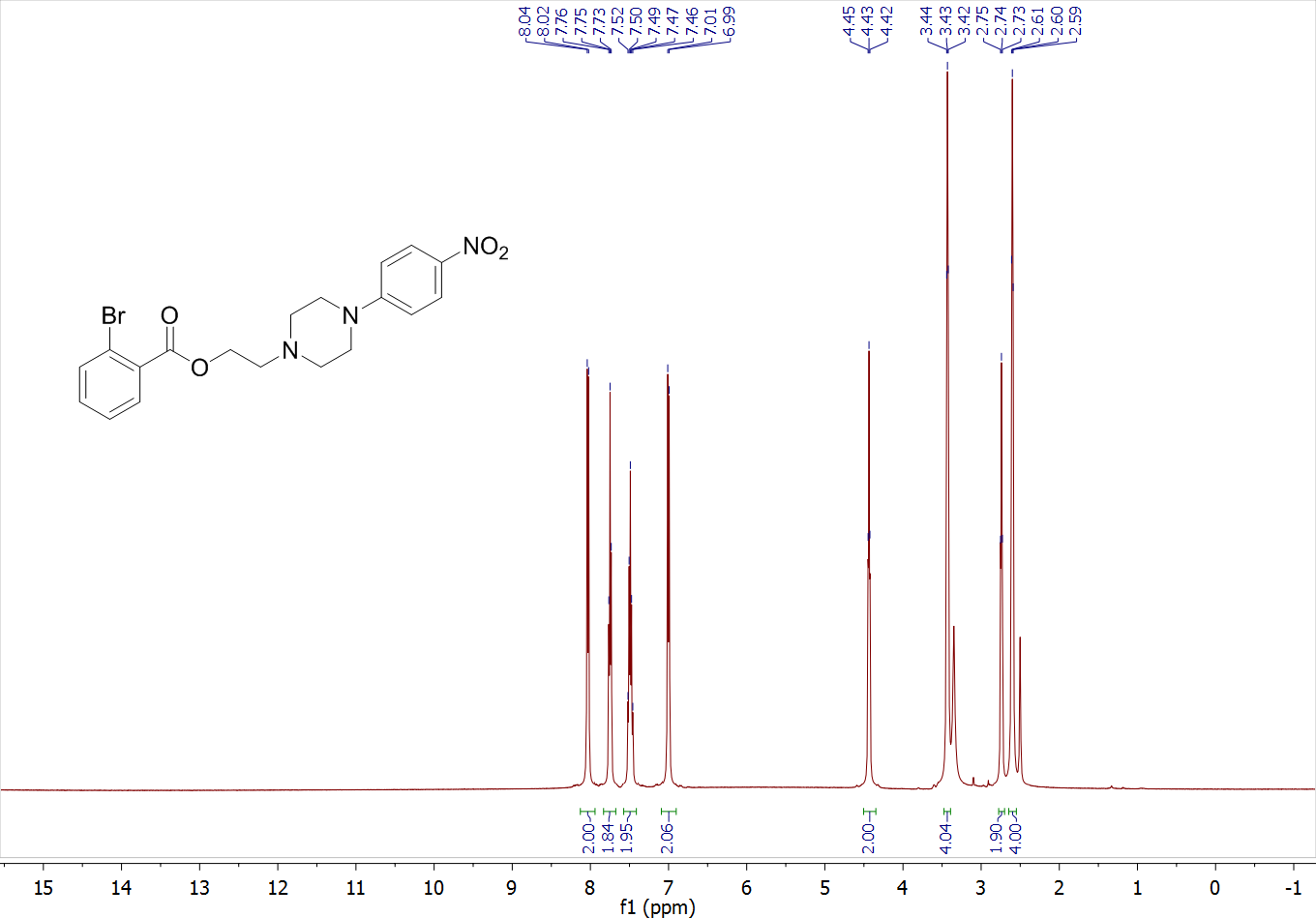


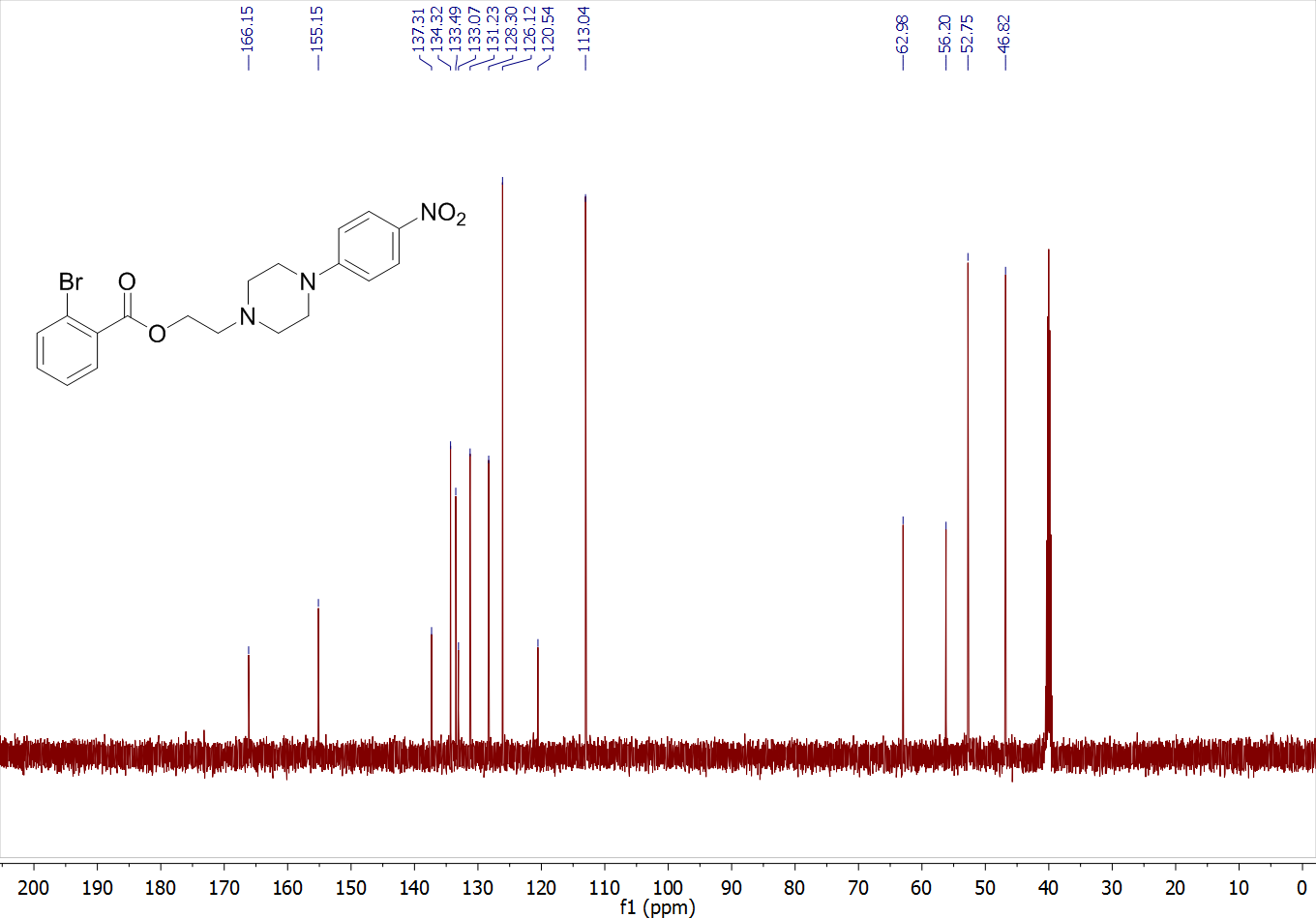


**2-(4-(4-nitrophenyl)piperazin-1-yl)ethyl 2,4-dichlorobenzoate (4c)**


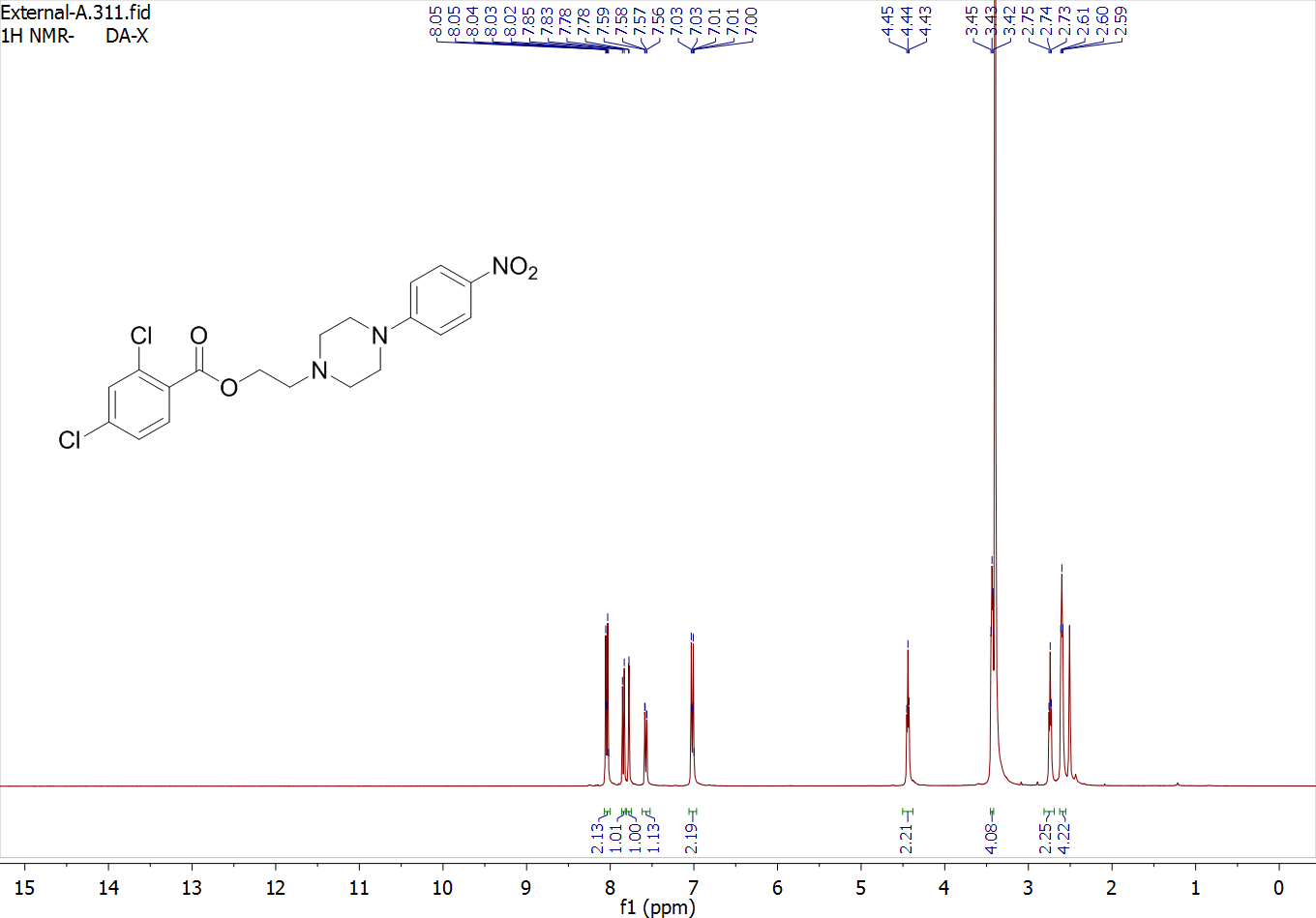


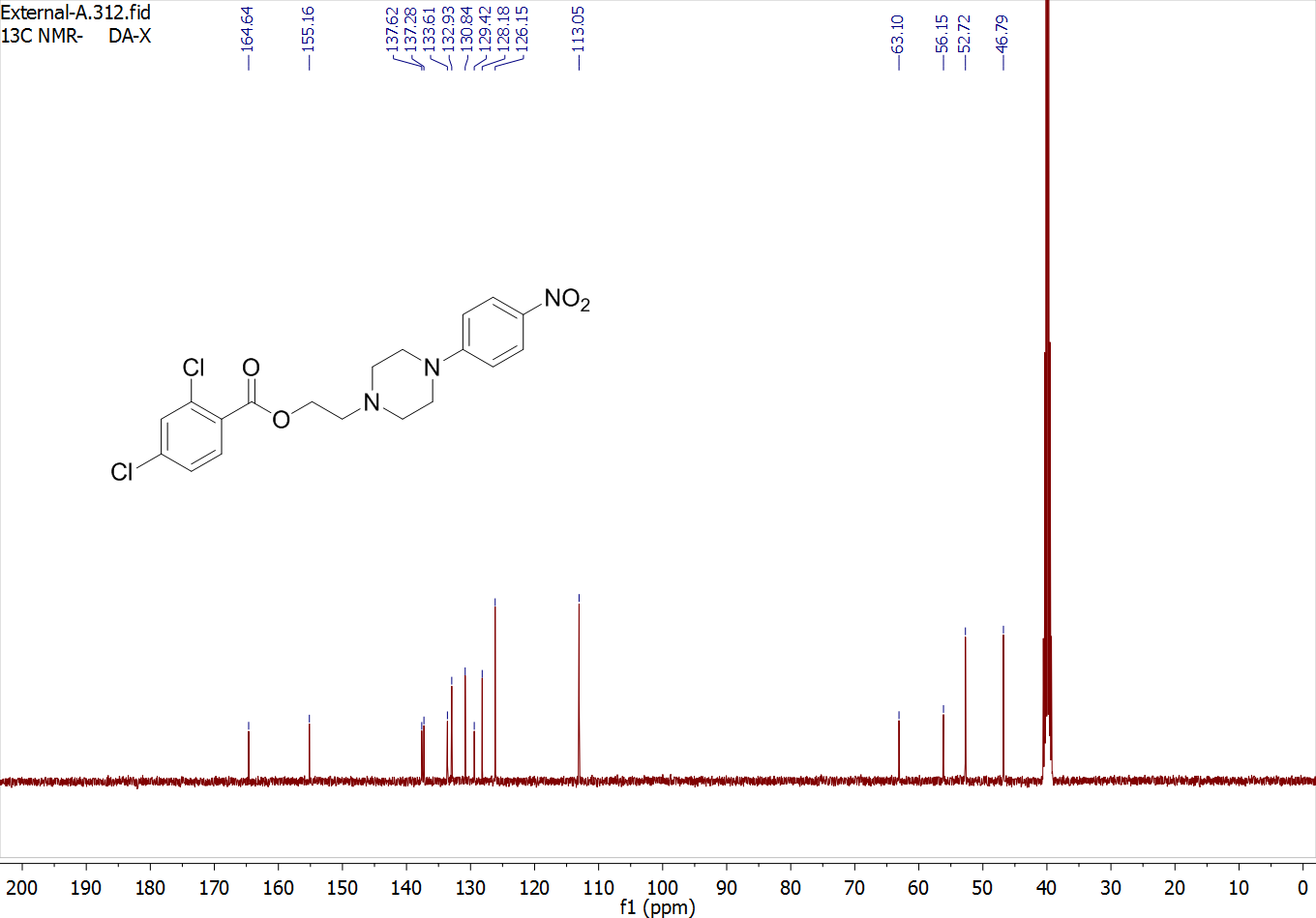


**2-(4-(4-nitrophenyl)piperazin-1-yl)ethyl 4-nitrobenzoate (4d)**


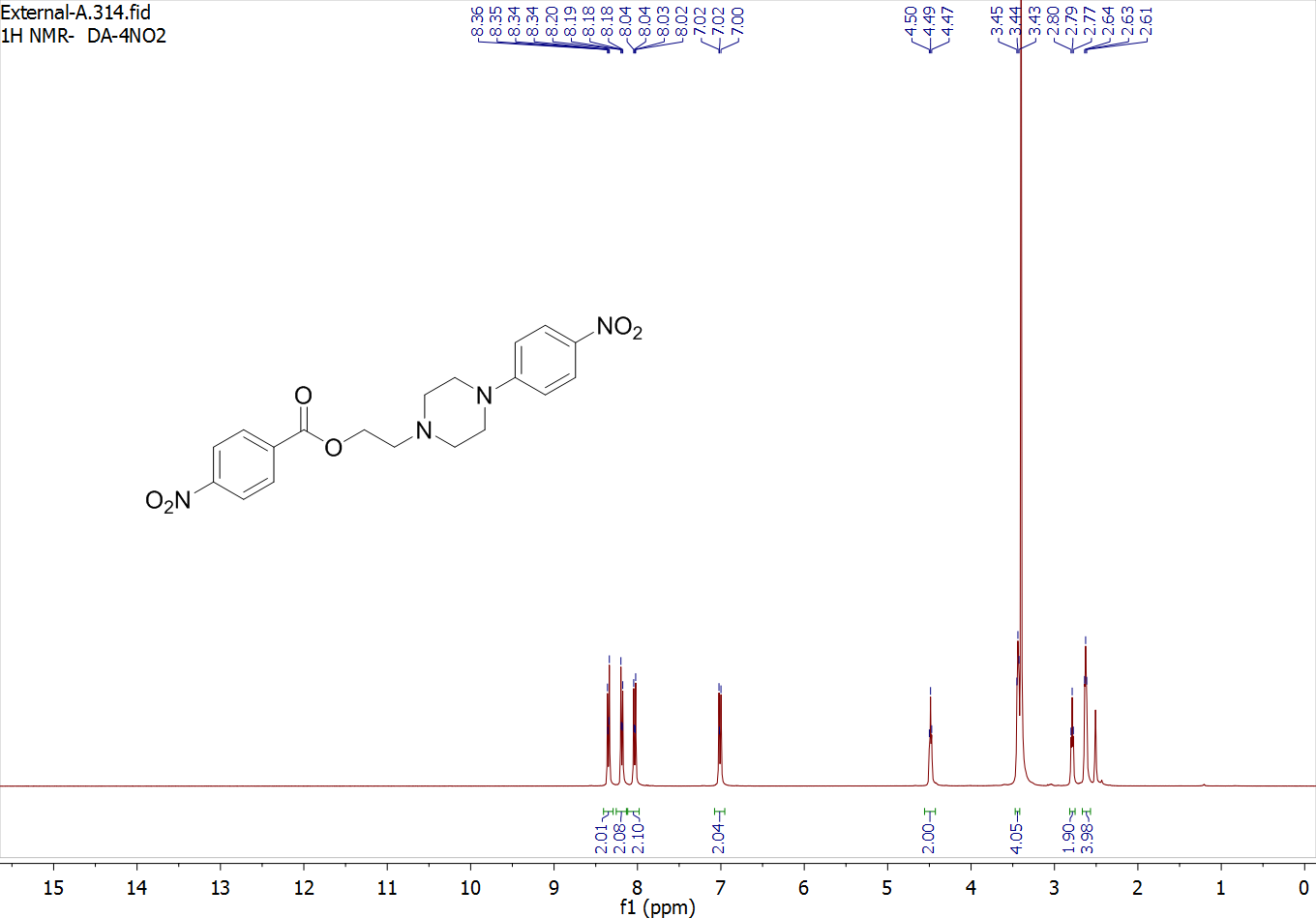


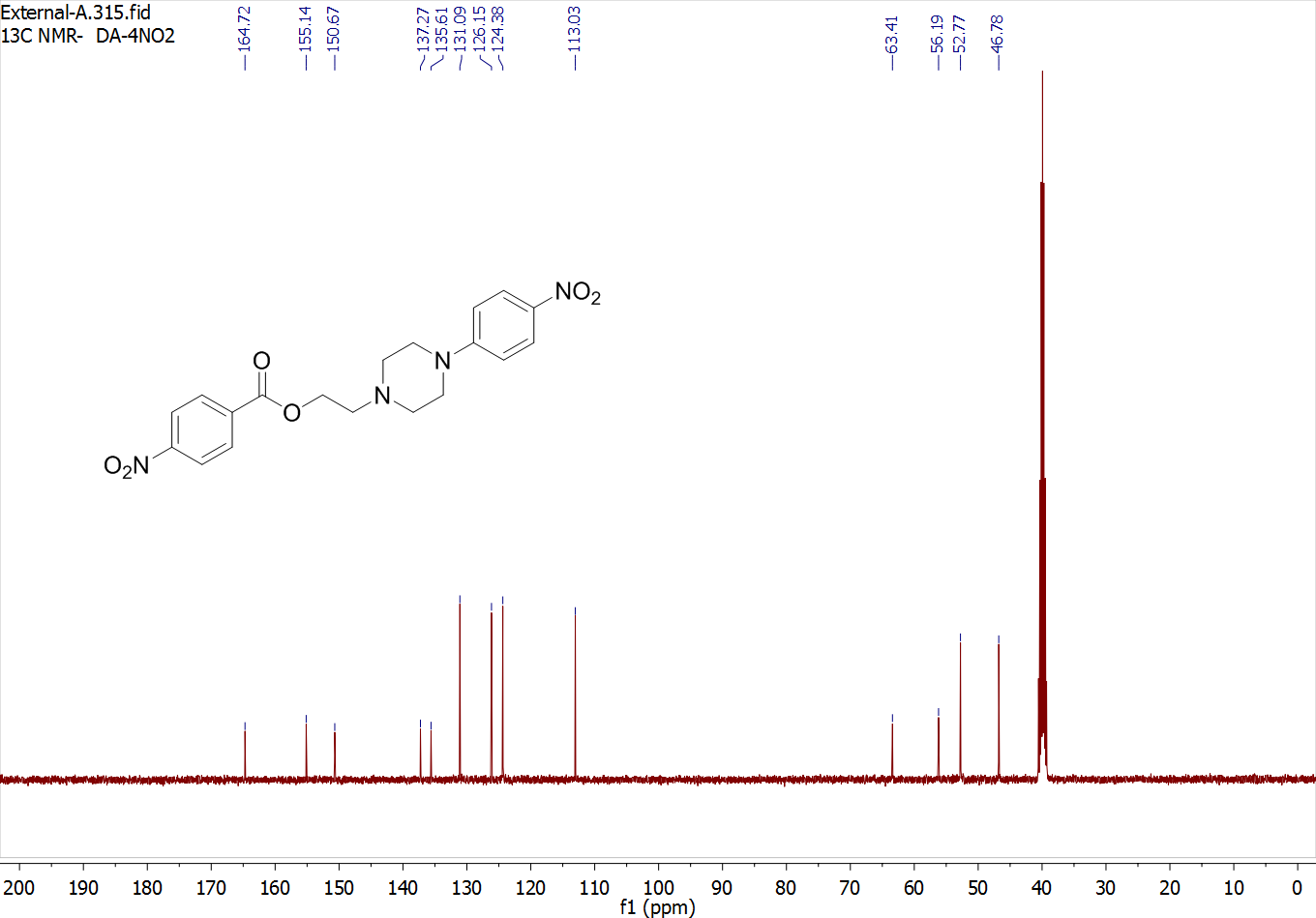


**2-(4-(4-nitrophenyl)piperazin-1-yl)ethyl 3-nitrobenzoate (4e)**


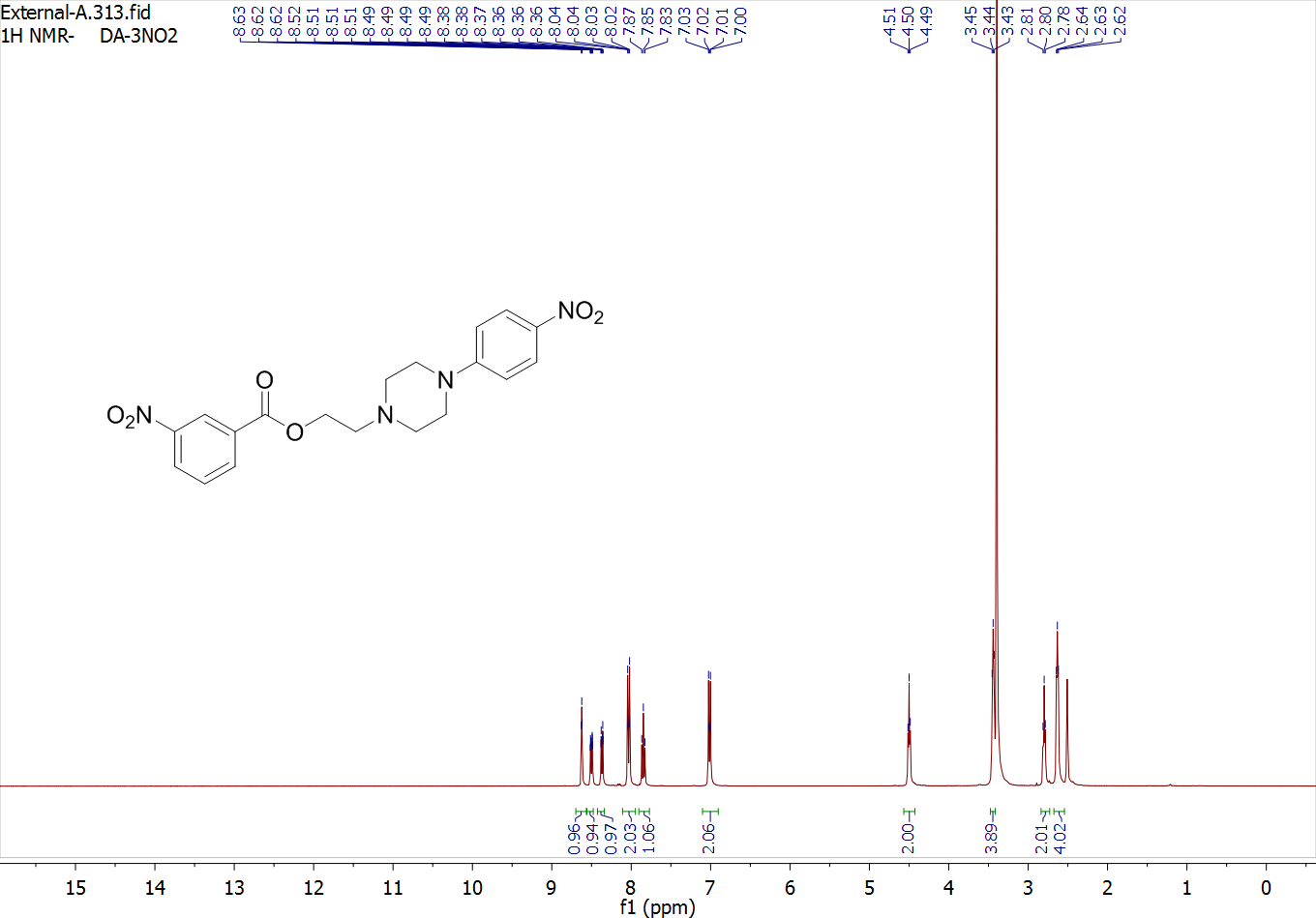


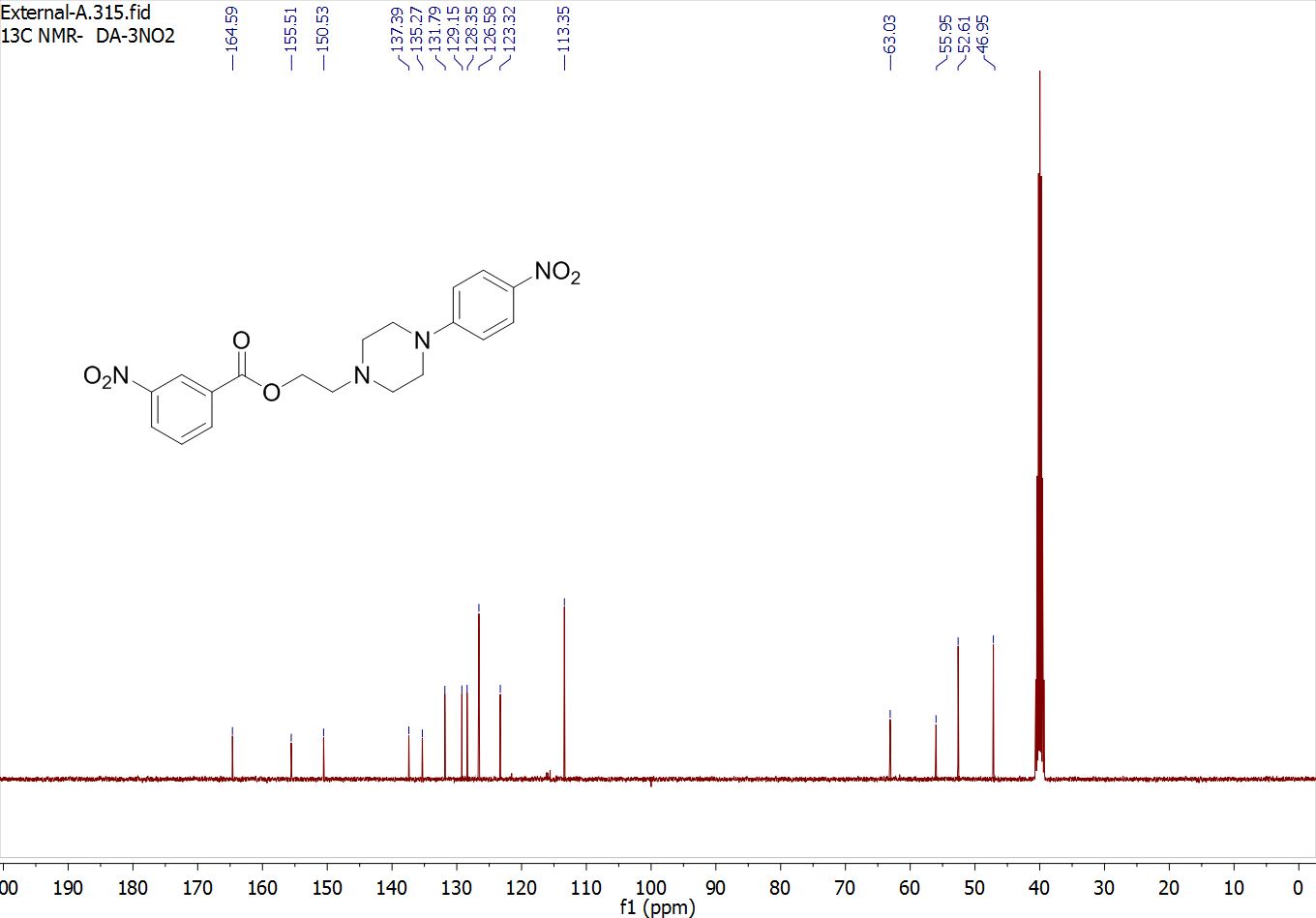


**2-(4-(4-nitrophenyl)piperazin-1-yl)ethyl 2-chloro-4-nitrobenzoate (4f)**


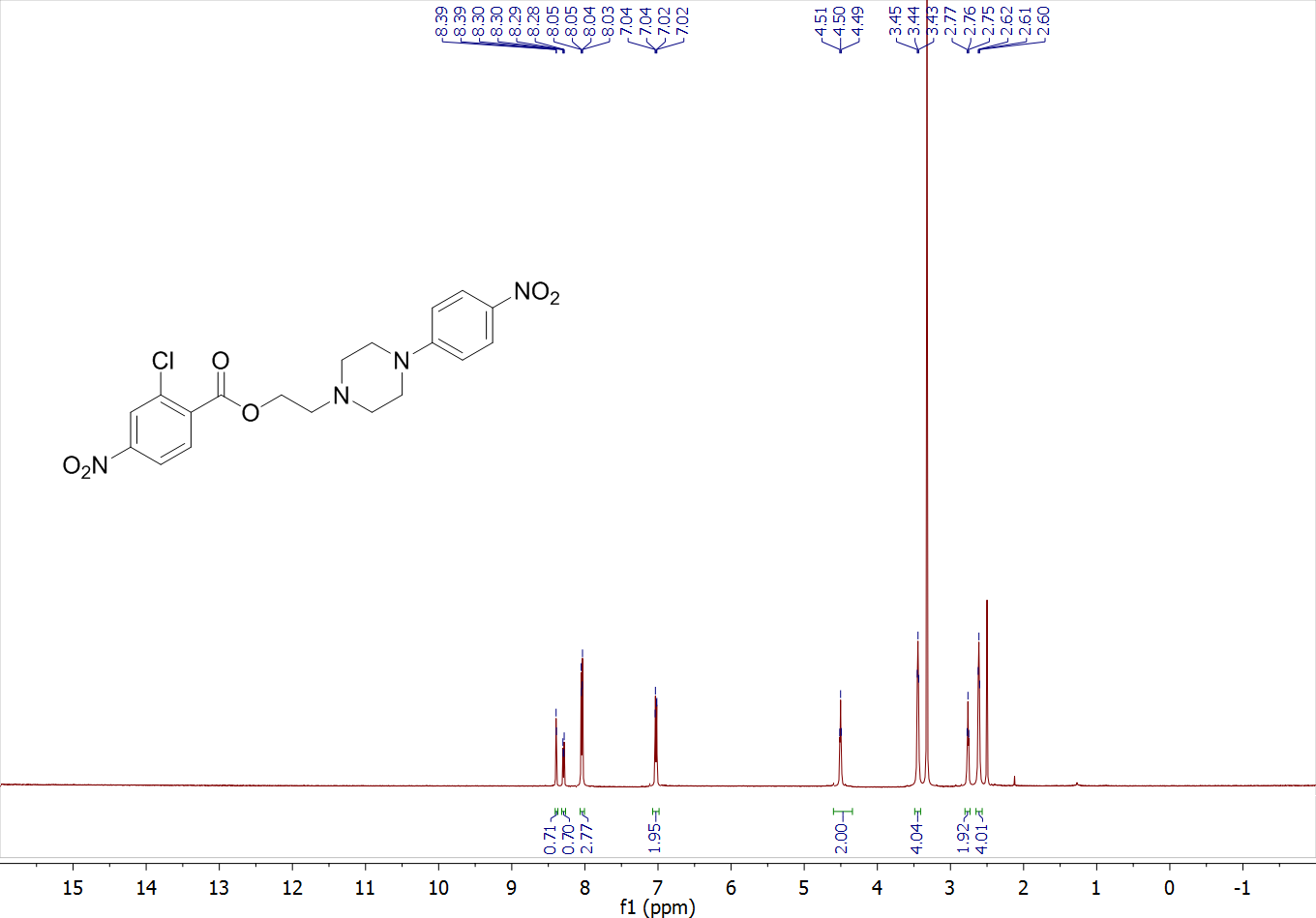


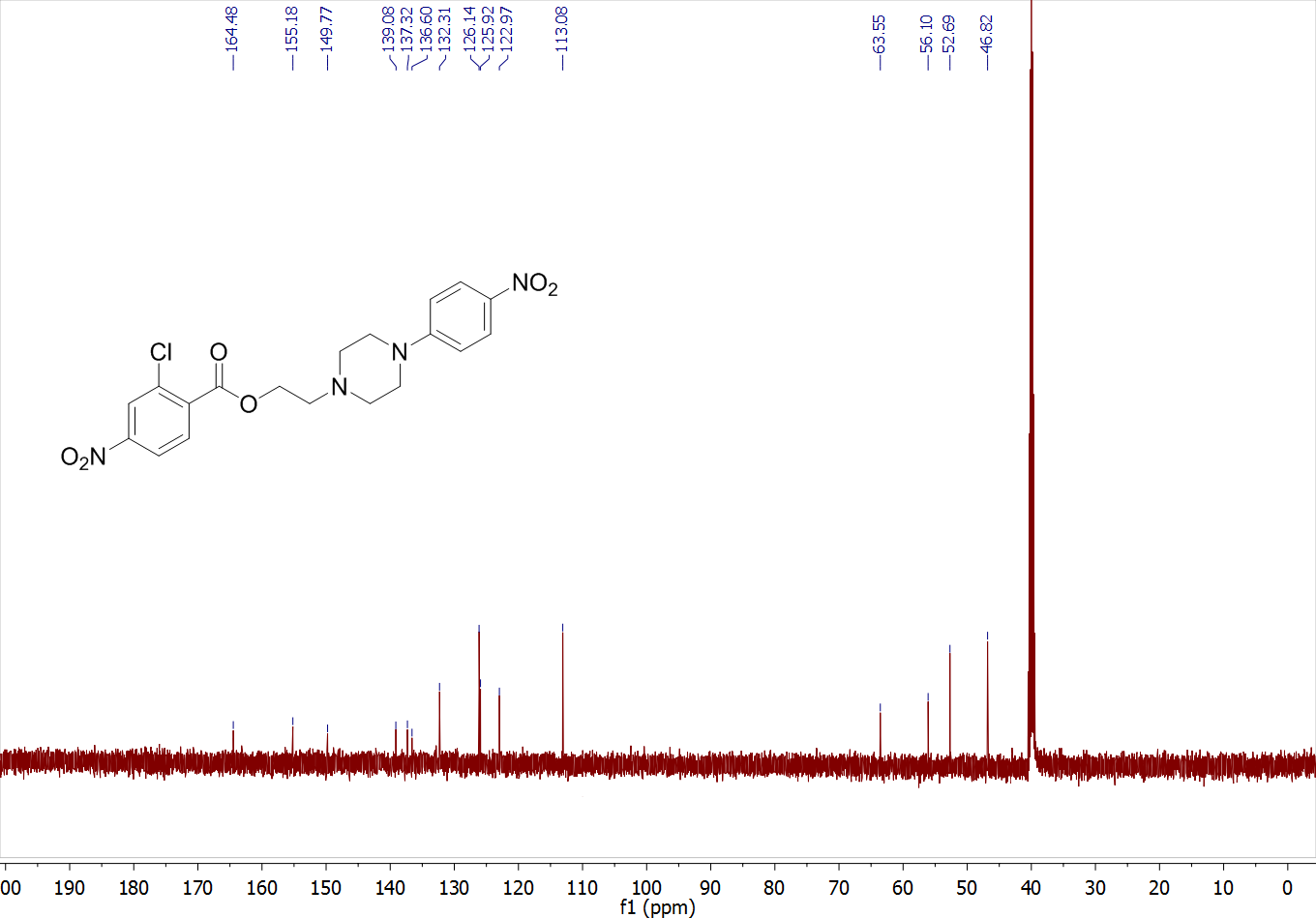


**2-(4-(4-nitrophenyl)piperazin-1-yl)ethyl 2-(4-methoxyphenyl)acetate (4g)**


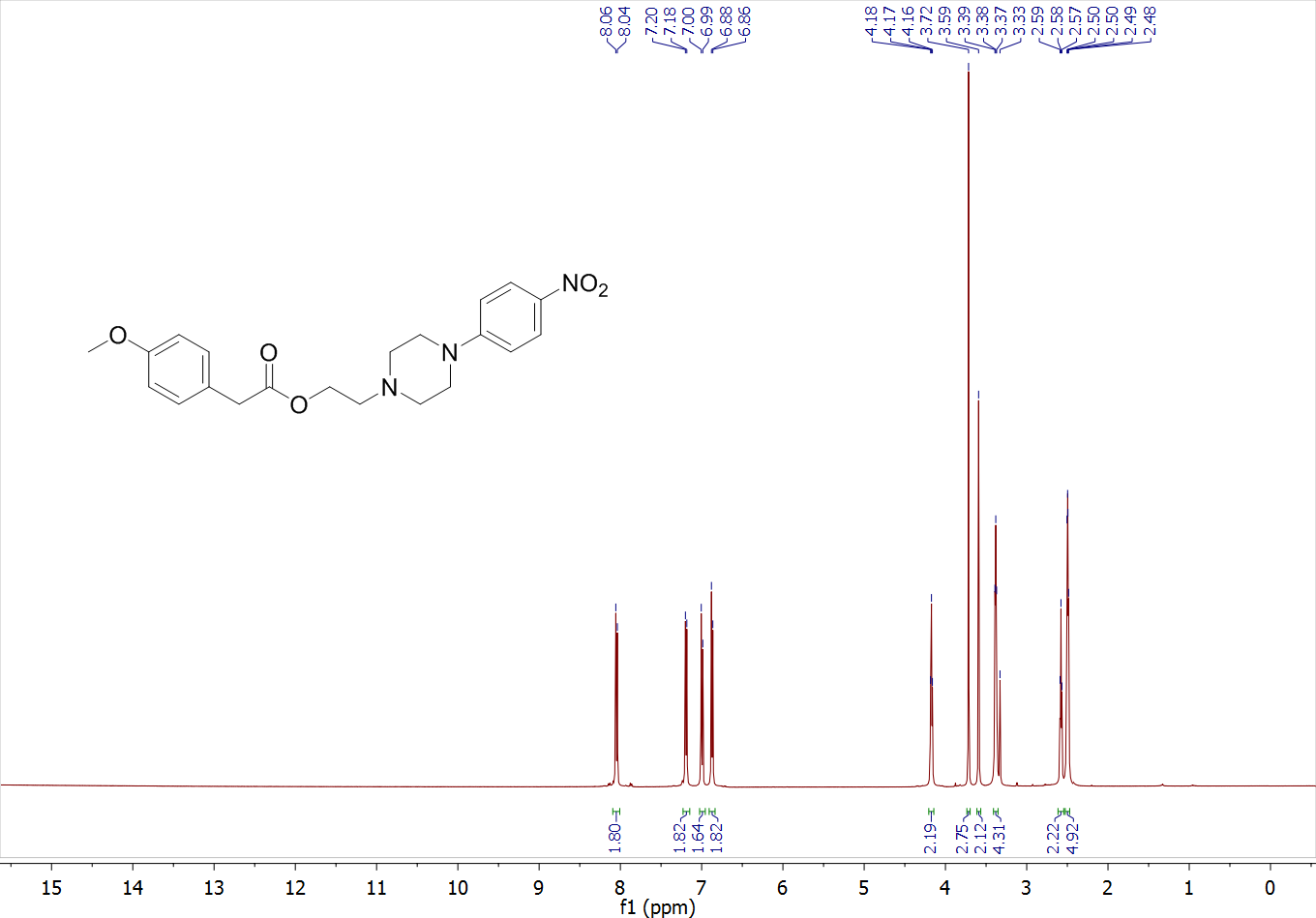


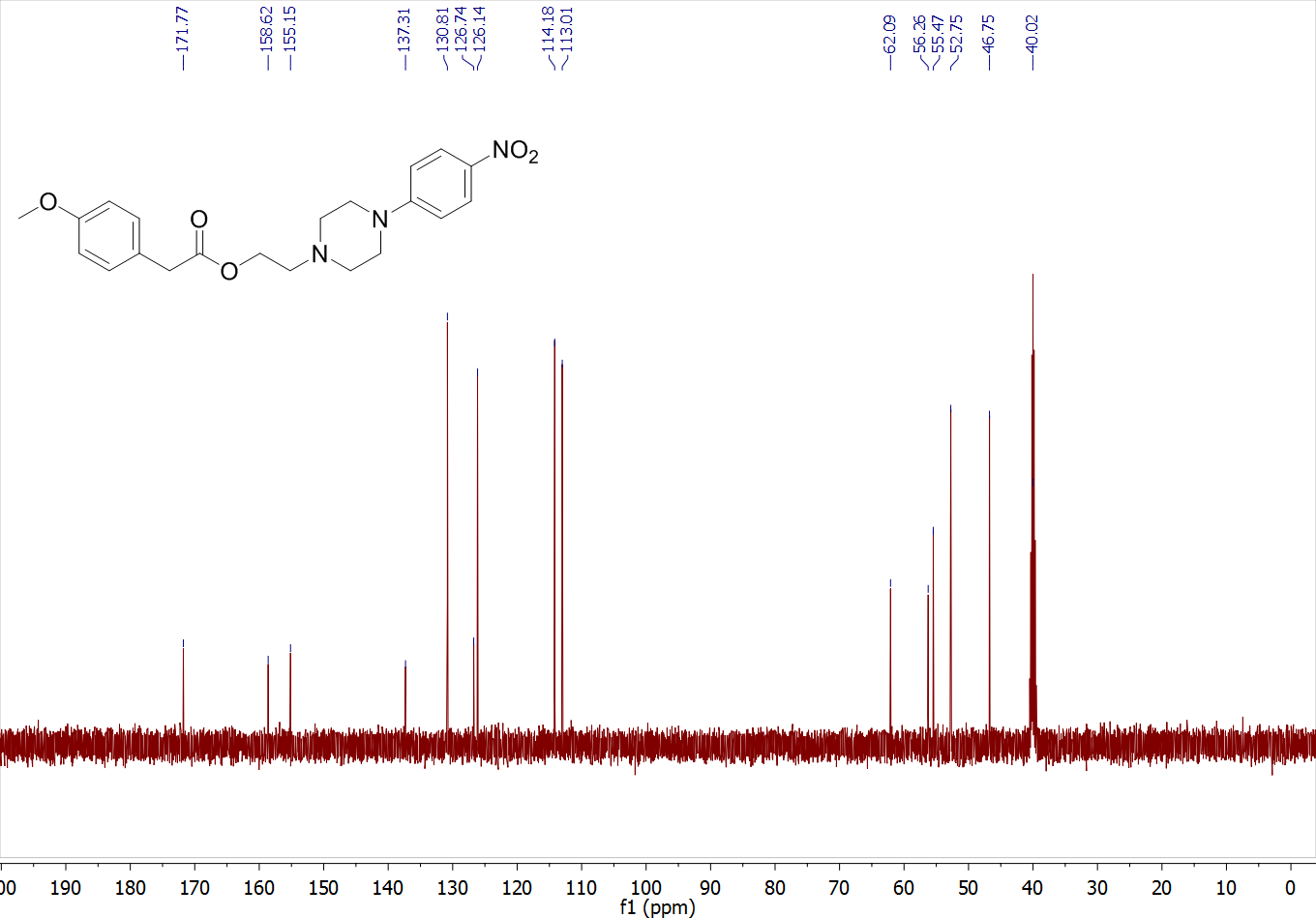


**2-(4-(4-nitrophenyl)piperazin-1-yl)ethyl 6-formyl-2,3-dimethoxybenzoate (4h)**


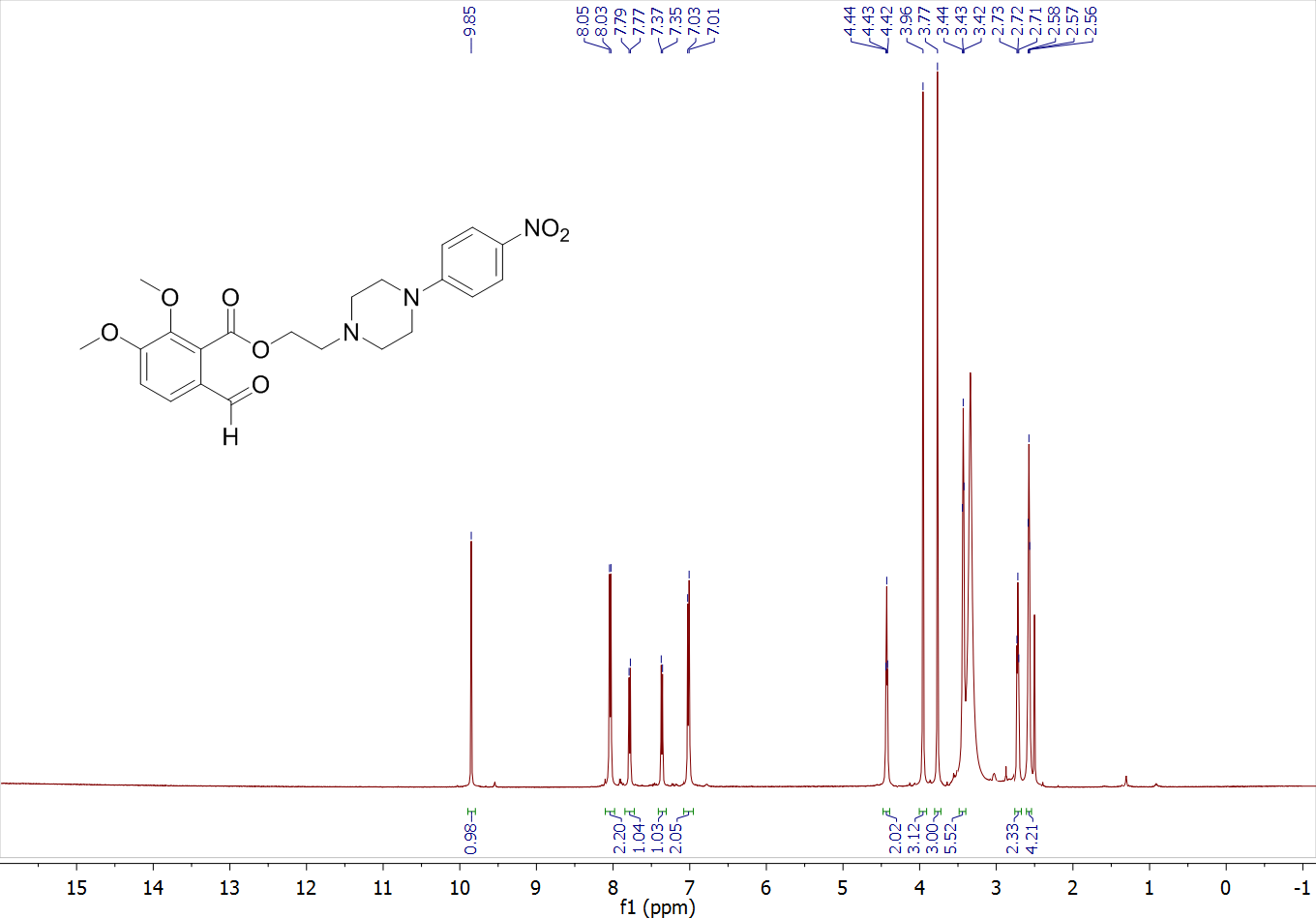


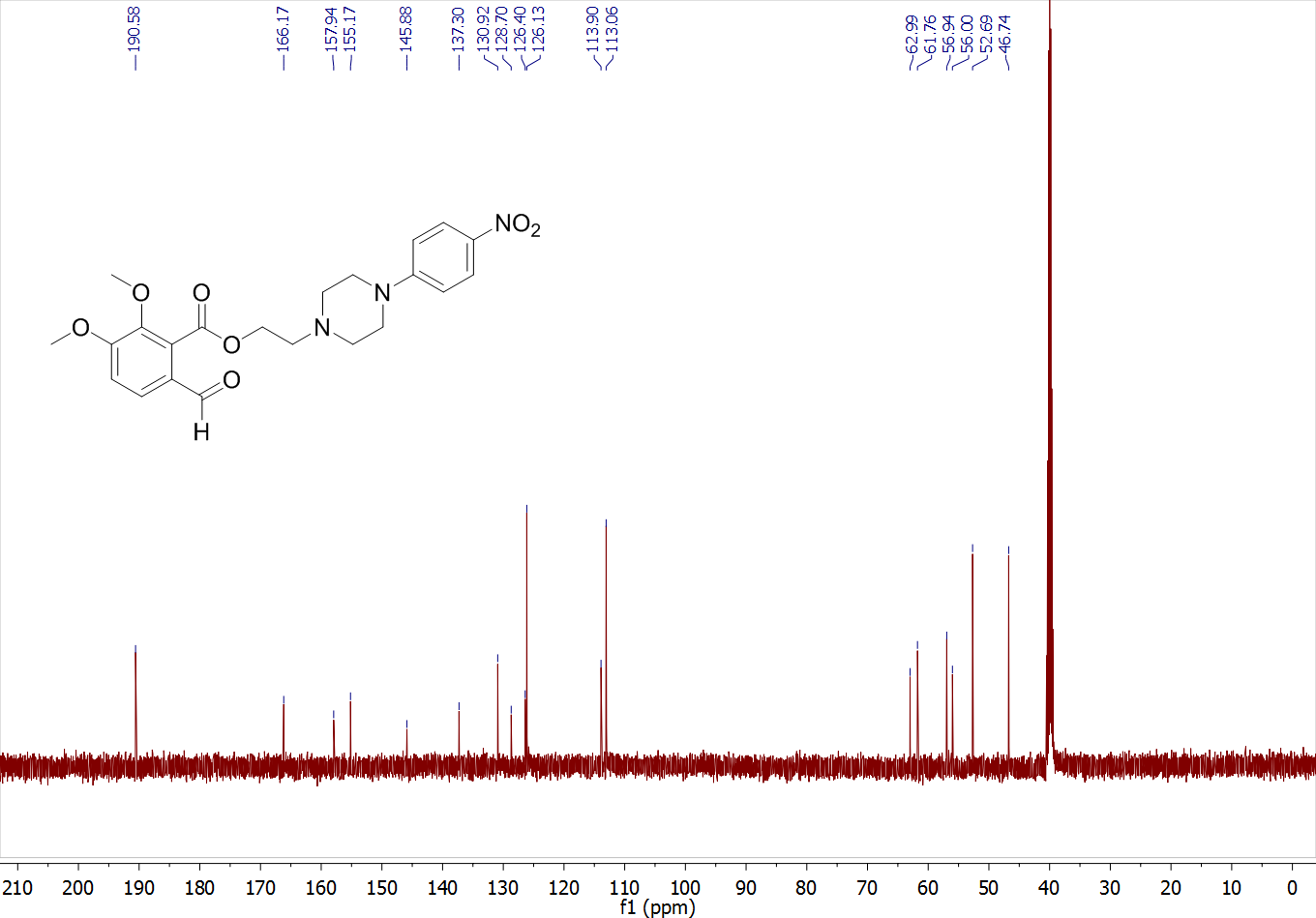


**2-(4-(4-nitrophenyl)piperazin-1-yl)ethyl 2-phenylacetate (4i)**


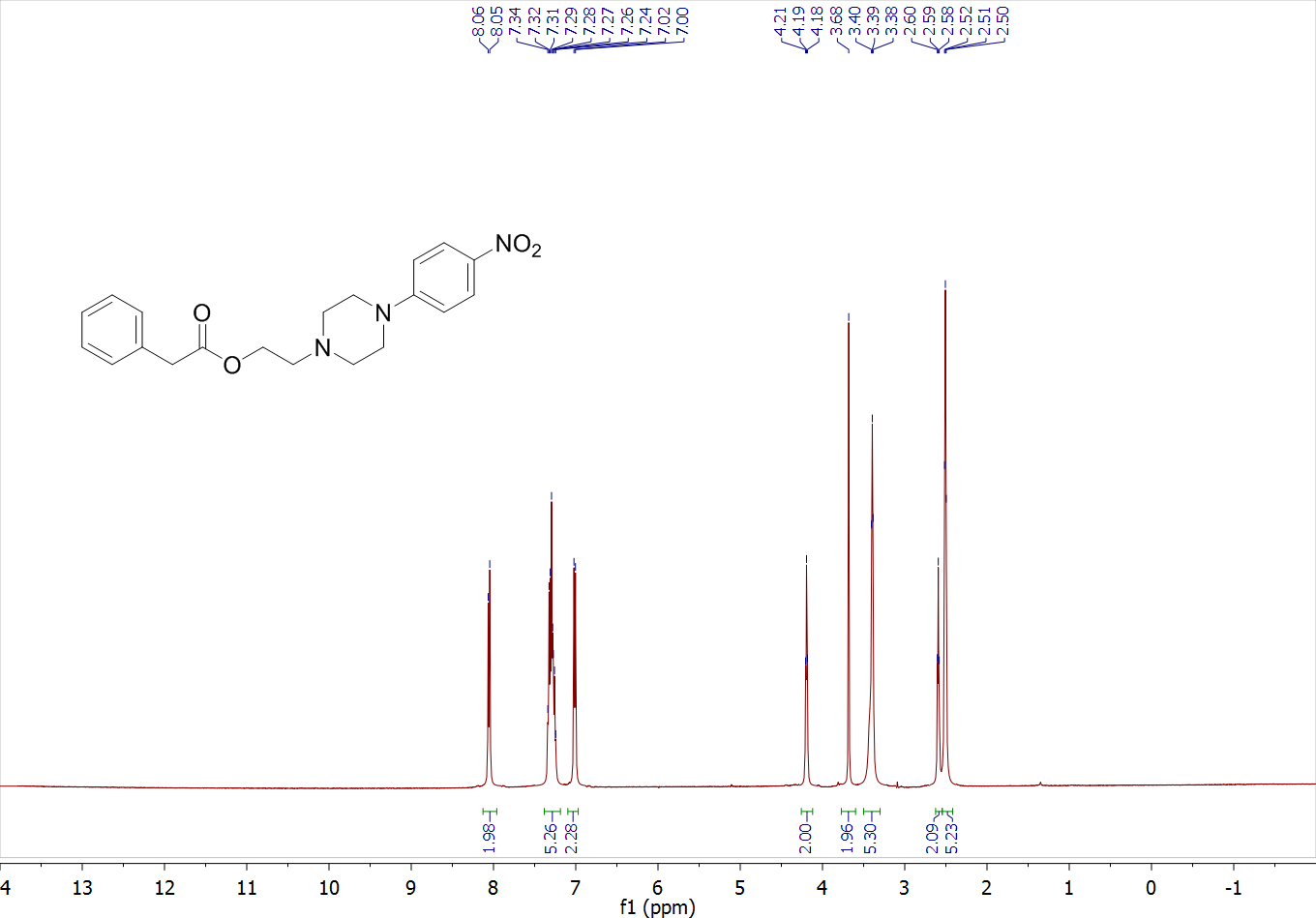


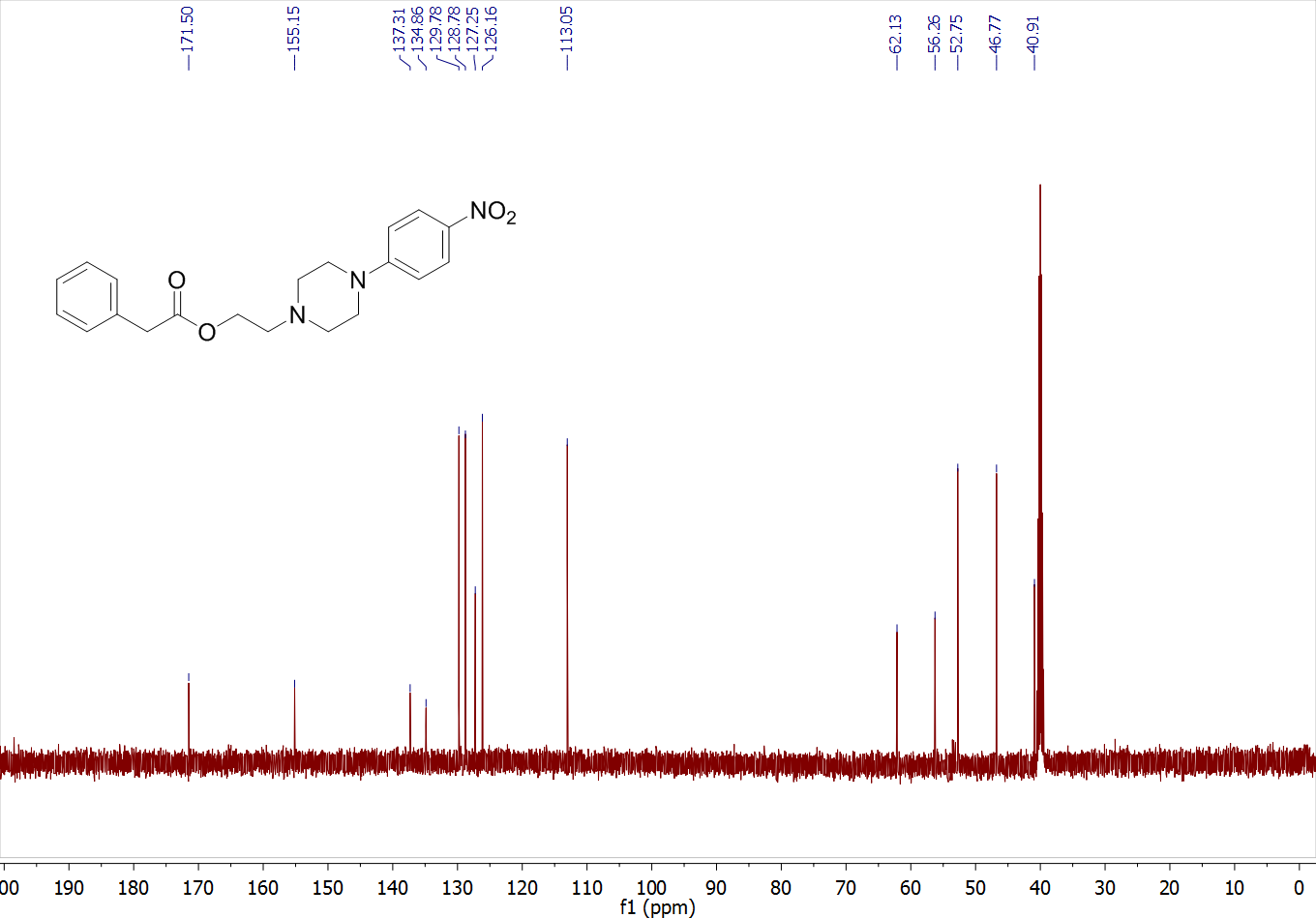


**2-(4-(4-nitrophenyl)piperazin-1-yl)ethyl cinnamate (4j)**


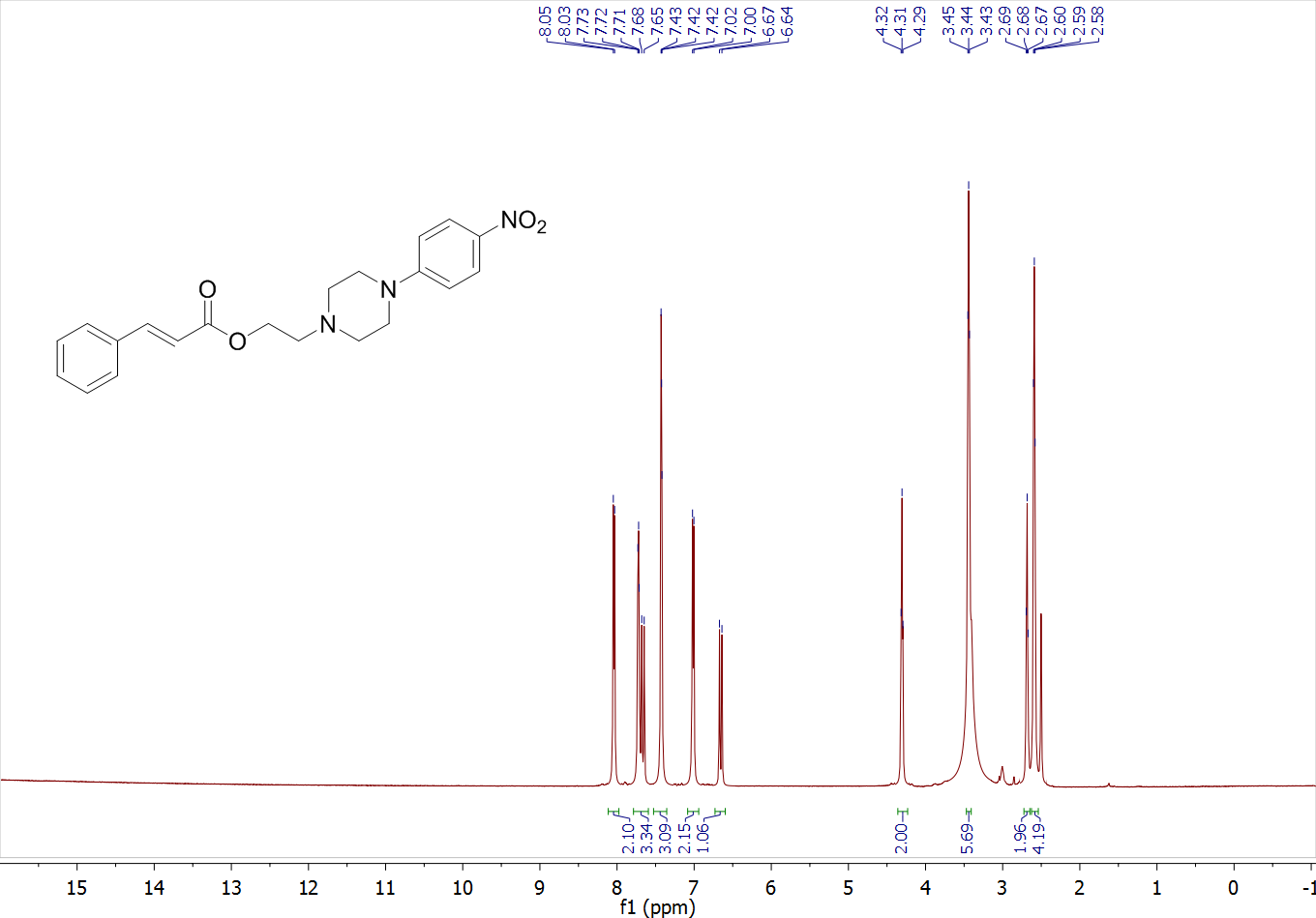


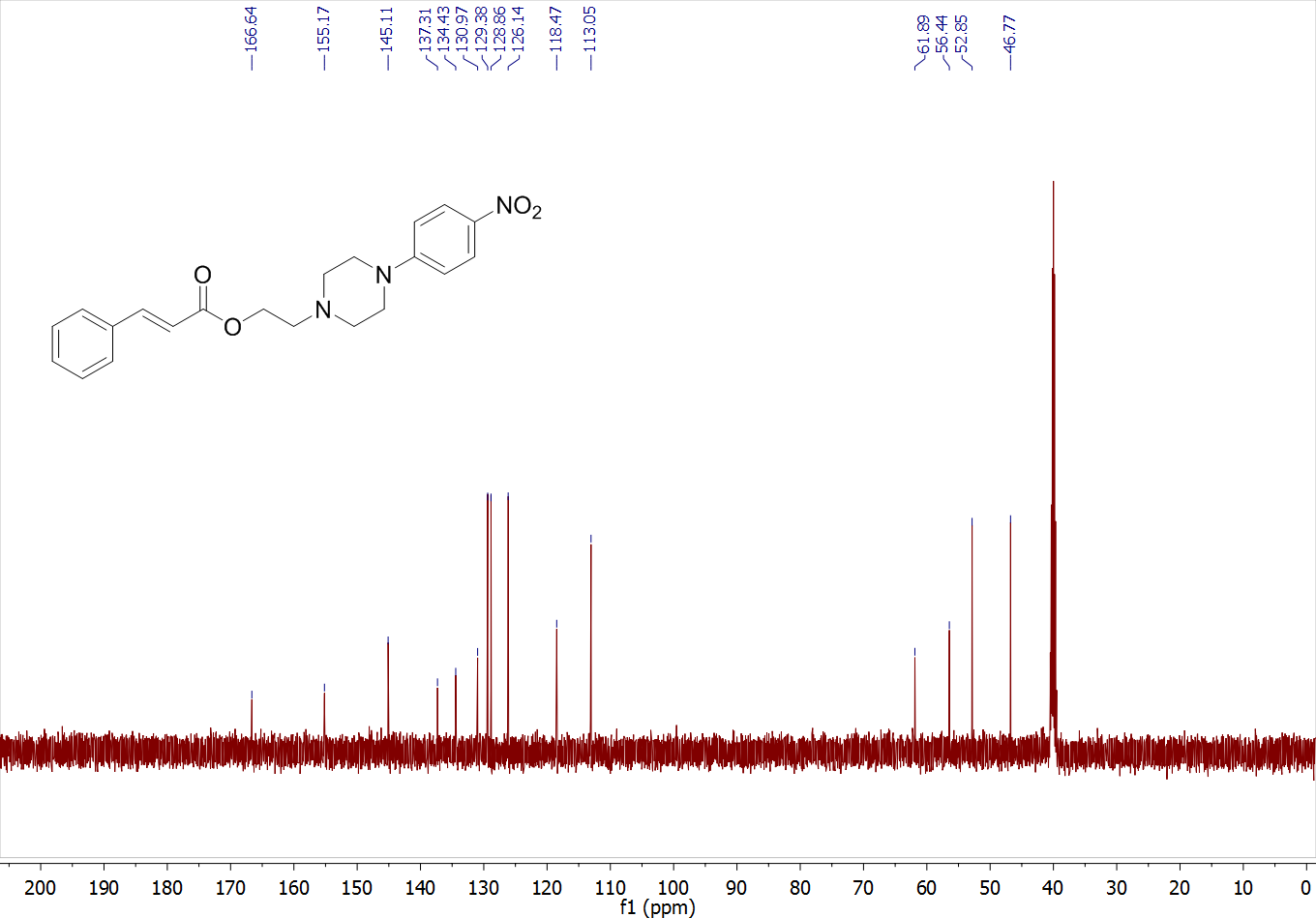


**2-(4-(4-nitrophenyl)piperazin-1-yl)ethyl nicotinate (4k)**


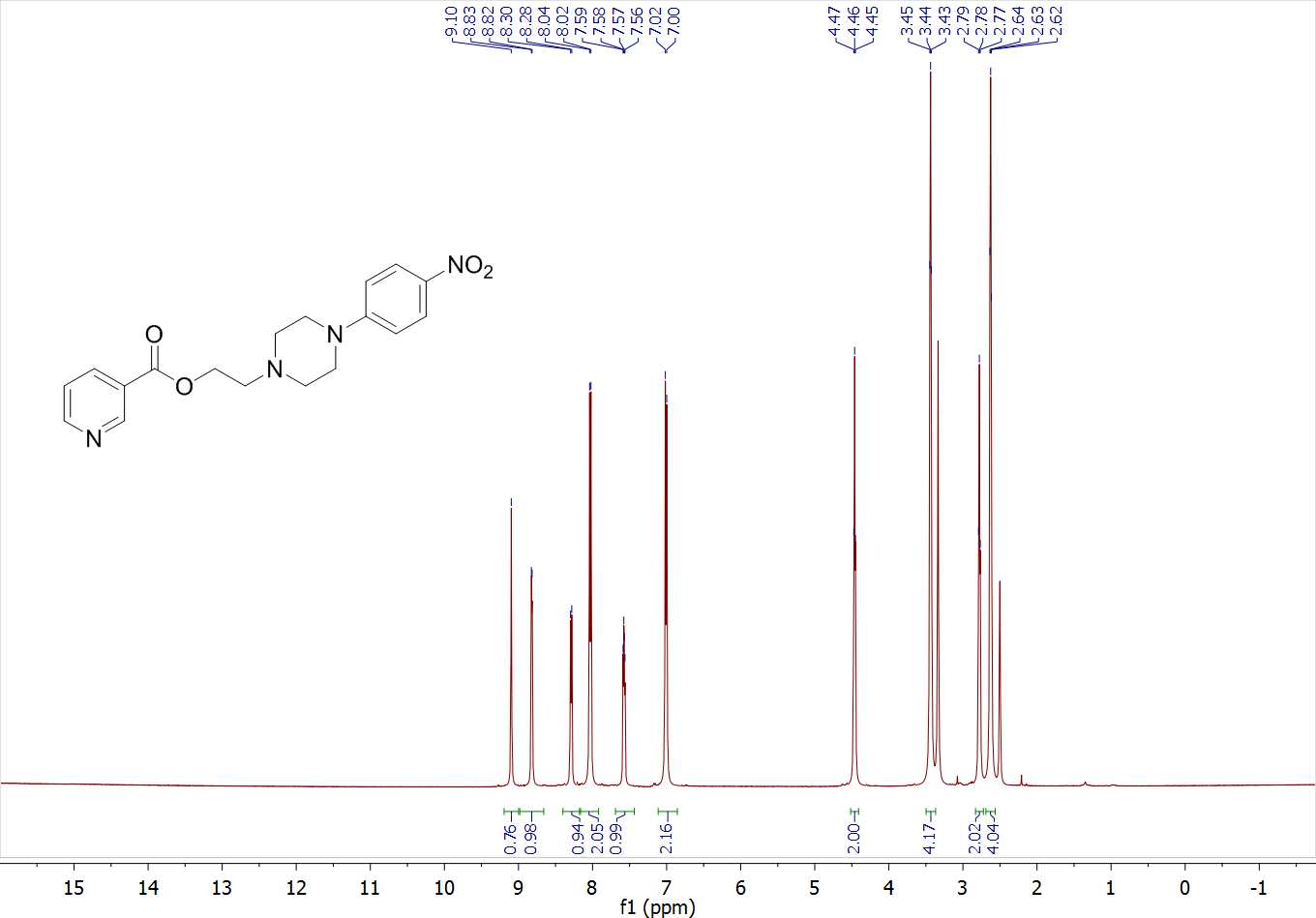


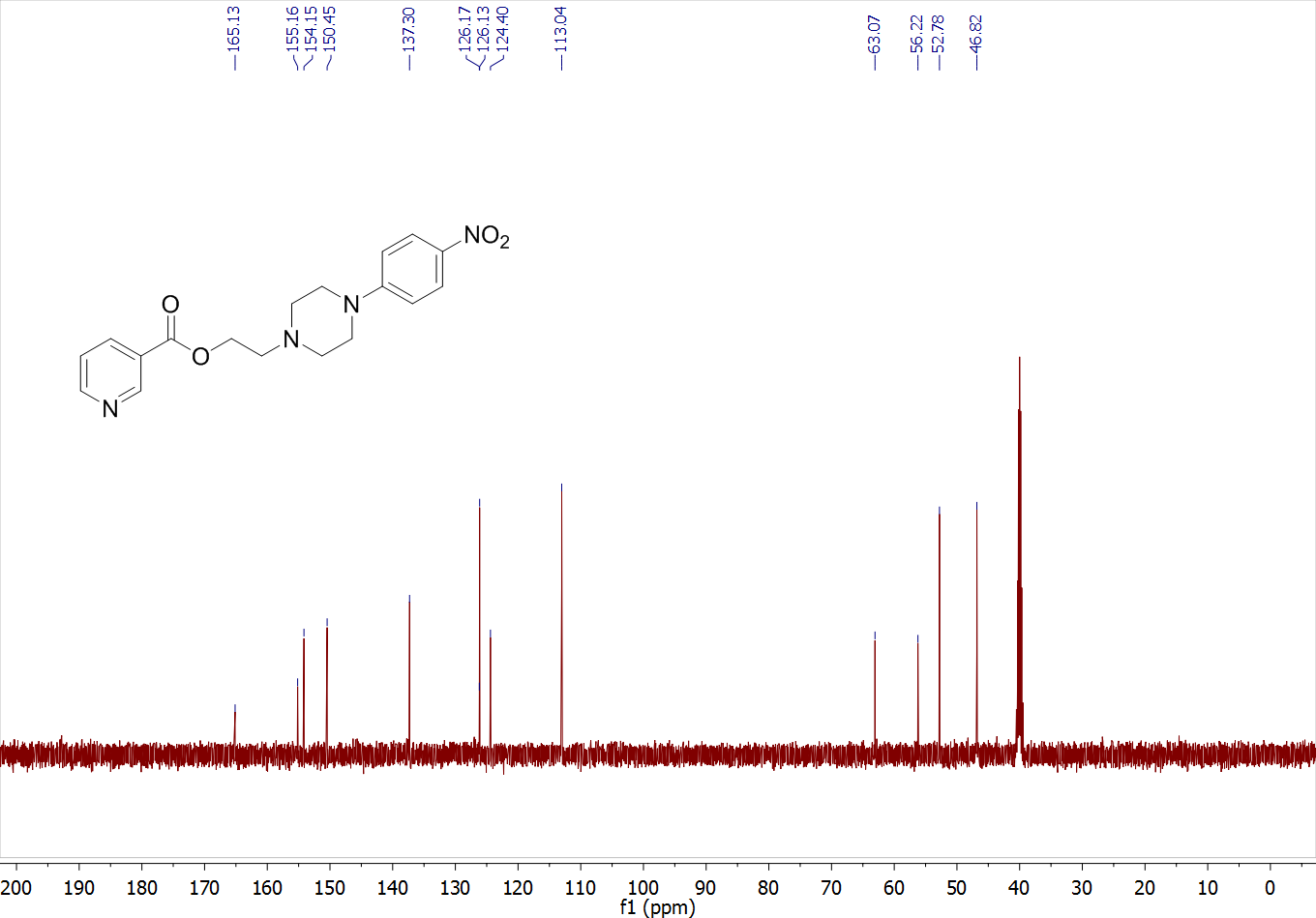


**2-(4-(4-nitrophenyl)piperazin-1-yl)ethyl 1*H*-indole-2-carboxylate (4l)**


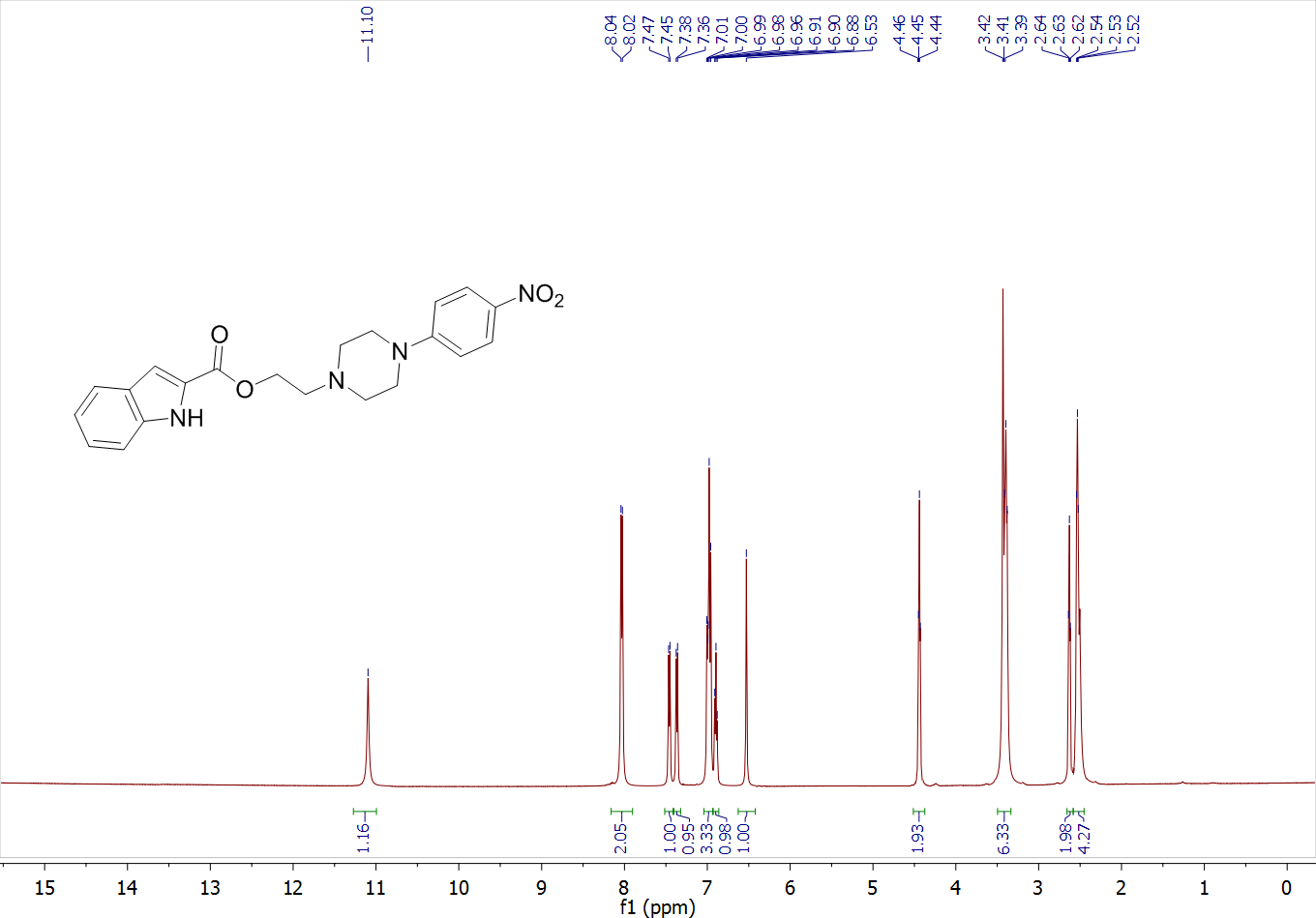


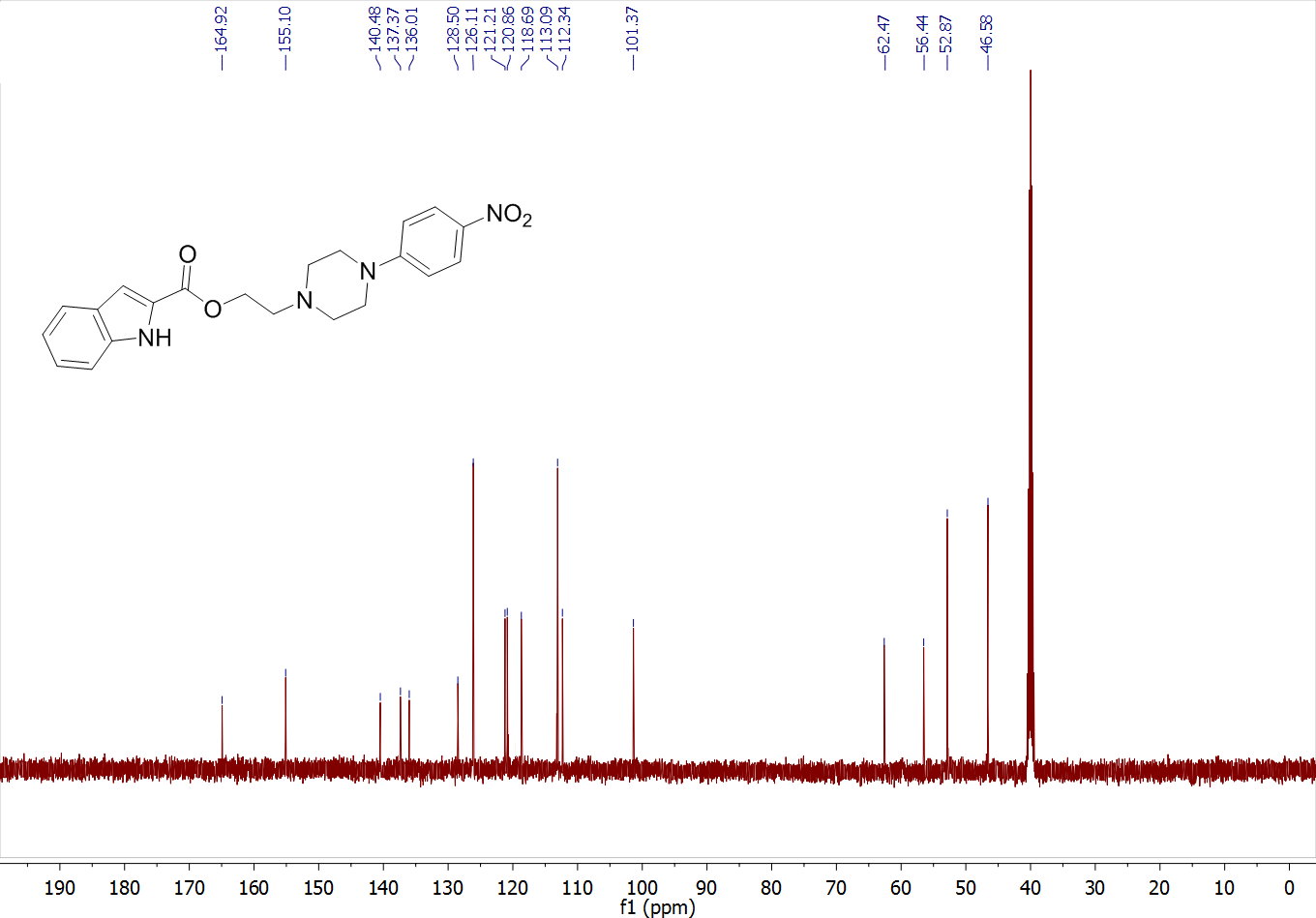


**2-(4-(4-nitrophenyl)piperazin-1-yl)ethyl 5-nitrofuran-2-carboxylate (4m)**


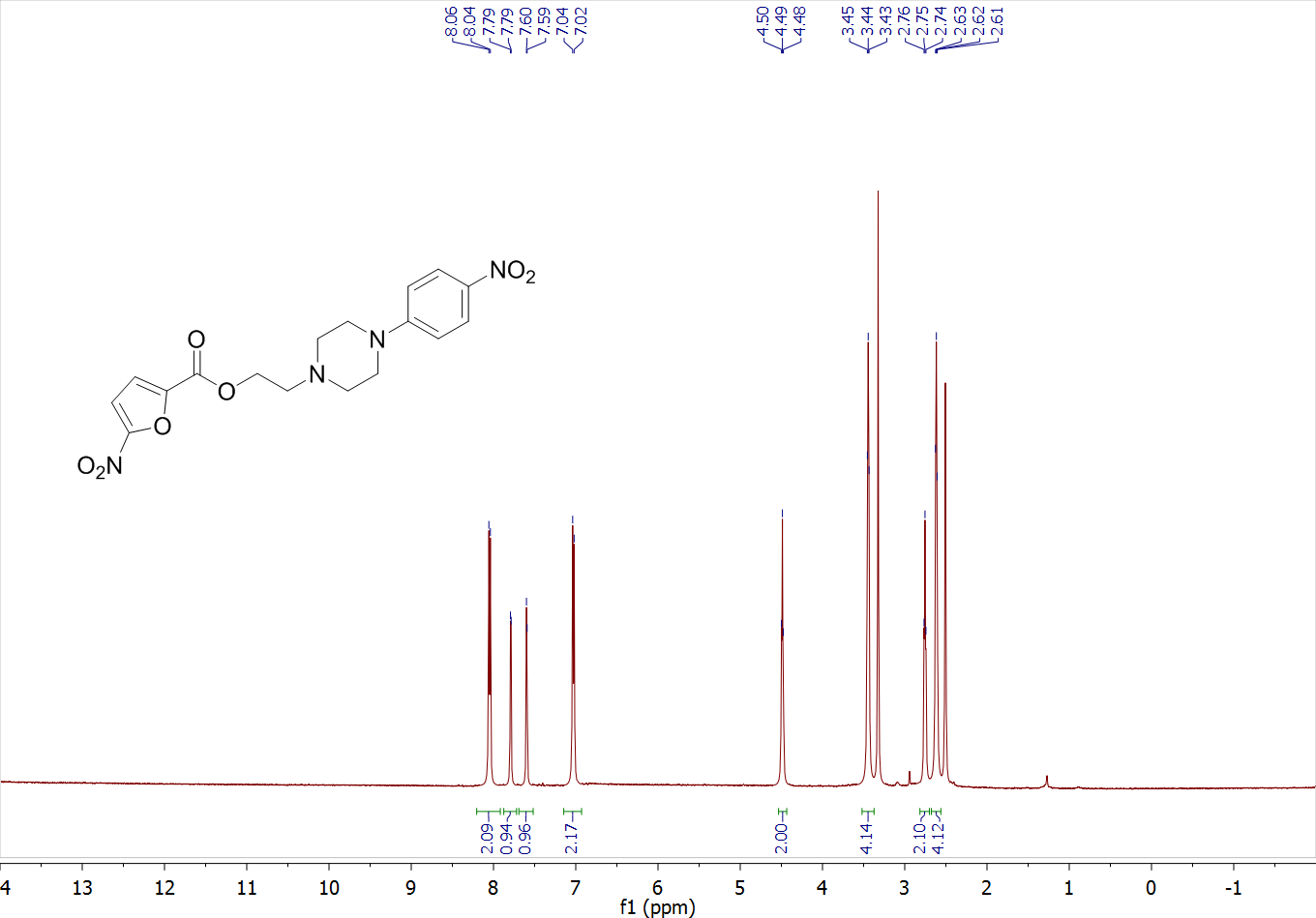


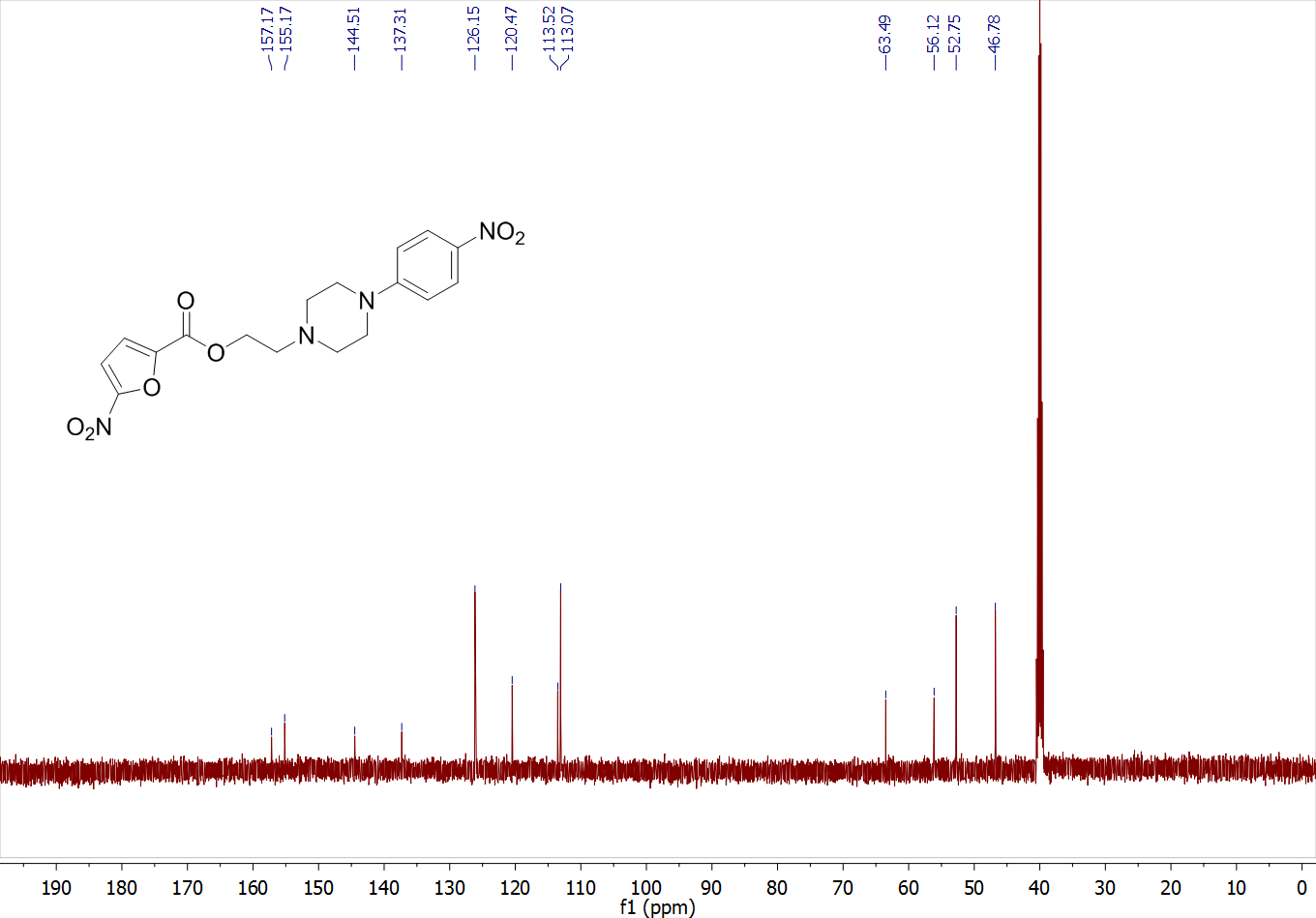

Supplement: Supplementary file 1 — Additional file 1: The Supplementary Information file contains NMR spectra. [file 13065_2024_1167_MOESM1_ESM.docx]
